# Synthesis and Biological Evaluation of Cyclobutane-Based β3 Integrin Antagonists: A Novel Approach to Targeting Integrins for Cancer Therapy

**DOI:** 10.3390/cancers15164023

**Published:** 2023-08-08

**Authors:** Mark Sutherland, Andrew Gordon, Fatemah O. F. O. Al-Shammari, Adam Throup, Amy Cilia La Corte, Helen Philippou, Steven D. Shnyder, Laurence H. Patterson, Helen M. Sheldrake

**Affiliations:** 1 Institute of Cancer Therapeutics, University of Bradford, Bradford BD7 1DP, UK; 2 Leeds Institute of Cardiovascular and Metabolic Medicine, University of Leeds, Leeds LS2 9JT, UK

**Keywords:** integrin αvβ3, integrin αIIbβ3, metastasis, RGD mimetic, cyclobutane, drug discovery

## Abstract

**Simple Summary:**

The integrin family of cell surface proteins plays an important role in the development and spread of cancers. Therefore, drugs which inhibit integrins should make effective cancer treatments. Most potential drugs developed so far target a single integrin and have not proved effective at treating cancer in human studies. Our research aims to develop more effective drugs by targeting two related integrins. This paper describes how these potential drug molecules are made, allowing chemists to make better compounds in the future, and describes the anti-integrin effects of the new compounds. Together this information will lead to the future design and development of better anticancer drugs.

**Abstract:**

The Arg-Gly-Asp (RGD)-binding family of integrin receptors, and notably the β3 subfamily, are key to multiple physiological processes involved in tissue development, cancer proliferation, and metastatic dissemination. While there is compelling preclinical evidence that both αvβ3 and αIIbβ3 are important anticancer targets, most integrin antagonists developed to target the β3 integrins are highly selective for αvβ3 or αIIbβ3. We report the design, synthesis, and biological evaluation of a new structural class of ligand-mimetic β3 integrin antagonist. These new antagonists combine a high activity against αvβ3 with a moderate affinity for αIIbβ3, providing the first evidence for a new approach to integrin targeting in cancer.

## 1. Introduction

The integrin family of cell surface glycoproteins control cell–extracellular matrix adhesion and signalling across the cell membrane. Functional integrins are present on the cell surface as heterodimers made up of an α and a β subunit; combinations of 18 α and 8 β subunits provide the 24 integrin receptors present in humans [1,2]. Integrins have a wide range of physiological functions, and a number of them have therefore gained considerable interest as drug targets [3,4]. The β3 integrin subfamily comprises two members: αIIbβ3 is normally found only on platelets where it mediates platelet cross-linking in the blood clotting process [5,6]. αvβ3 is most highly expressed on endothelial cells, controlling cell survival and signalling pathways that regulate angiogenesis [7], and thus has become an attractive target for disorders involving neoangiogenesis.

Changes in integrin expression are associated with cancer development and progression, and in particular, the abnormal expression and activity of β3 integrins in tumour cells is associated with cancer progression and metastasis [8,9]. The expression of αvβ3 is strongly associated with advancing disease and poor prognosis. It promotes cell survival [10,11,12], migration [12,13,14], and metastasis via the lymph system [15] and bloodstream [13,14] and is particularly important in the development and growth of bone metastases [16,17,18,19,20,21,22,23,24]. αvβ3 is a stem cell marker [25], and promotes resistance to a number of cytotoxic and targeted chemotherapy agents [26]. The ectopic expression of αIIbβ3 is associated with increased tumour growth and metastatic disease [27,28,29,30,31,32,33]. Haematogenous metastasis is promoted by the interaction of tumour cells and platelets mediated by tumoural β3 integrins and platelet αIIbβ3, resulting in platelet activation and aggregation, the release of growth factors, and increased cell survival in the blood stream in addition to adhesion [34] and invasion at the metastatic site [32,35,36,37,38]. αIIbβ3 antagonists have been shown to be effective in reducing the metastasis of melanoma and breast cancer cells [22,39,40,41]. In cells expressing both β3 integrins, αIIbβ3 can supplant and suppress αvβ3 function [29], suggesting these tumours will be resistant to selective αvβ3 antagonists.

Despite promising preclinical results of β3 antagonists as anticancer agents, the failure of the first-in-class αvβ3 antagonist cilengitide to meet its primary endpoint in Phase II and III clinical trials has discouraged further exploration of αvβ3-targeted anticancer agents [42,43]. A number of reasons for failure have been proposed. αvβ3 antagonists have shown partial agonism and paradoxical effects at low concentrations [44,45], so the rapid clearance of cilengitide in vivo may result in an ineffective target coverage and partial promotion of tumour growth. Closer attention to pharmacokinetics and dosing schedules may be required for successful integrin-targeted therapy [46].

The development of αIIbβ3 antagonists has been similarly challenging. Initial successes with tirofiban, eptifibatide and abciximab as antithrombotic agents in the acute hospital setting encouraged the development of other small molecules. However, multiple failures in clinical trials led to their development being discontinued. Like αvβ3, αIIbβ3 antagonists are liable to paradoxical effects [47]. As antiplatelet agents, they are also prone to bleeding side-effects [48]. However, some studies indicate this is not inevitable [49,50] or can be mitigated by an appropriate dosing strategy [48].

Small molecule β3 antagonists have traditionally been designed to be selective for either αIIbβ3 or αvβ3. For example, cilengitide has a high affinity for αvβ3 but substantially lower anti-αIIbβ3 activity [51]. We have rationalised that dual αIIbβ3/αvβ3 antagonists will have superior anticancer effects due to their ability to antagonise multiple mechanisms involved in tumour cell survival and dissemination and have specific utility in treating tumours characterised by the expression of both β3 integrins or haematogenous metastasis [8]. β3 downregulation, suppressing the expression of both αvβ3 and αIIbβ3 integrins, significantly inhibits tumour growth, invasion, recurrence, and metastasis [14,52,53,54]. Studies with monoclonal anti-αIIbβ3/αvβ3 antibodies, or combinations of selective antagonists, have shown that dual β3 antagonism is effective at blocking tumour growth and angiogenesis through targeting tumour cell interaction with platelets and endothelial cells as well as tumour tissue [55,56,57,58], and is more effective than the use of a single integrin-targeted agent [59]. As a high expression of the β3 integrins is a particular feature of melanoma, the dual antagonist approach may be particularly valuable in treating advanced or high-risk melanomas.

We have recently developed efficient and scalable routes to highly functionalised four-membered rings [60,61], structures that to date have been underexploited in drug design despite their potential for metabolic stability and predictable pharmacokinetics [62,63]. We predicted that these cyclobutanes would possess suitable conformational and pharmacokinetic properties for use as the core scaffold in Arg-Gly-Asp (RGD)-mimetic integrin antagonists controlling the orientation of Arg and Asp mimetic sidechains presented to the integrin. A range of molecules were designed to explore the effects of sidechain identity, orientation, and length on anti-integrin activity with the aim of identifying a safe and effective dual β3 integrin antagonist. This paper describes the synthesis and initial investigation of the biological activity of cyclobutane antagonists employing pyrimidine, naphthyridine, or tetrahydronaphthyridine (THN) groups as the arginine mimetic.

## 2. Materials and Methods

### 2.1. General

Chemical reagents and anhydrous solvents were obtained from Sigma-Aldrich (Poole, Dorset, UK) and used without further purification. All other solvents were supplied by VWR (Poole, UK). Unless otherwise stated, reactions were carried out in anhydrous solvent and were not air-sensitive. Petroleum ether (PE) refers to the fraction boiling between 60 and 80 °C. Flash chromatography was carried out on silica gel (Merck 9385 Kieselgel 60 (230–400 ASTM) (VWR) or Davisil 60 A, 40–63 μm (Fisher Scientific, Loughborough, UK). Analytical TLC was carried out on 0.25 mm thick aluminium plates precoated with Merck Kieselgel F_254_ silica gel (VWR) and visualised by UV and aqueous alkaline potassium permanganate solution. Preparative TLC was carried out on Analtech silica plates with UV245 indicator (Sigma-Aldrich). NMR spectra were recorded on a Jeol GX270 or Bruker DPX400 spectrometer (Bruker, Coventry UK). Multiplets are indicated as: s singlet; d doublet; t triplet; q quartet; qn quintet; dd double doublet; dt double triplet; m multiplet; br broad; app apparent. Melting points were determined using a Gallenkamp melting point apparatus (VWR) and are uncorrected.

Sources of biological reagents are specified in each protocol. RGDS was obtained from Sigma-Aldrich, cRGDfV from Enzo Life Sciences (Farmingdale, NY, USA), and GR144053 from Tocris Bioscience (Bristol, UK).

Human melanoma SK-Mel-2 and M14 cells (ATCC, LGC Standards, Teddington, UK) were cultured in RPMI 1640 cell culture medium supplemented with 10% FBS, 1 mM sodium pyruvate and 2 mM L-glutamine (all Sigma) at 37 °C in a humidified atmosphere containing 5% CO_2_. Cells were not used for more than 10 passages.

### 2.2. Molecular Modelling

The distance between Arg and Asp mimetic sidechains was measured on the minimum energy conformation of the molecule after the molecular geometry was optimised in Arguslab using the PM3 Hamiltonian and a maximum number of steps set to 10,000. Docking studies were carried out using the standard Arguslab docking protocol [64] with Protein Data Bank crystal structures 1TY5 (αIIbβ3) and 1L5G (αvβ3). Ligand groups were created from the previously minimised structure of the compound to be docked and the original ligand (tirofiban or cilengitide, respectively) present in the PDB crystal structure, and the binding site defined by creating a binding-site group from the original ligand. After docking, the 5 lowest energy poses were reviewed.

### 2.3. Chemical Synthesis

#### 2.3.1. 4-(1,3-Dioxo-1,3-dihydro-isoindol-2-yl)-butyraldehyde **1**

To a stirred solution of 4-chloro-1-butanol (1.012 g, 9.32 mmol) in DMF (5 mL) was added potassium phthalate (1.727 g, 9.32 mmol) and the reaction mixture heated to 150 °C for 23 h. The reaction mixture was poured into water (50 mL) and extracted with EtOAc (5 × 20 mL). the combined organic layers were concentrated in vacuo and purified by flash column chromatography (EtOAc:PE, 3:7→1:1) to yield 2-(4-hydroxy-butyl)-isoindole-1,3-dione (960 mg, 47%) as oily yellow crystals. To a stirred solution of this alcohol (530 mg, 2.42 mmol) in DCM (10 mL) was added MgSO_4_ (10 g) and PCC (1.565 g 7.26 mmol), and the resulting suspension vigorously stirred for 1.75 h. The reaction mixture was filtered through 1 cm SiO_2_ washing with EtOAc and the filtrate concentrated in vacuo and purified by flash column chromatography (EtOAc:PE, 2:3→1:1) to yield the title compound (404 mg, 77%) as white crystals: *R_f_* 0.20 (EtOAc:PE, 3:7). ^1^H NMR (400 MHz, CDCl_3_) δ 9.77 (t, *J* = 1.0 Hz, 1H, H-1), 7.83–7.85 (m, 2H, Ar*H*), 7.71–7.73 (m, 2H, Ar*H*), 3.74 (t, *J* = 7.1 Hz, 2H, H-4), 2.54 (dt, *J* = 1.0, 7.1 Hz, 2H, H-2), 2.02 (qn, *J* = 7.1 Hz, 2H, H-3).

#### 2.3.2. 5-(1,3-Dioxo-1,3-dihydro-isoindol-2-yl)-pentanal **2**

Method of Sall [65]: A mixture of 5-aminopentanol (759 mg, 7.37 mmol) and phthalic anhydride 1.09 g, 7.37 mmol) was heated to 138 °C for 20 h. The resulting brown oil was cooled to room temperature and dissolved in DCM (35 mL). To the resulting solution was added MgSO_4_ (10 g) and PCC (4.77 g, 22.11 mmol) and the resulting suspension stirred at room temperature for 1 h 10 min. The reaction mixture was filtered through Celite™ washing with EtOAc. The filtrate was concentrated in vacuo and purified by flash column chromatography (EtOAc:PE, 2:3) to yield the title compound (1.41 g, 83%) as a colourless oil: *R_f_* 0.22 (EtOAc:PE, 3:7). ^1^H NMR (400 MHz, CDCl_3_) δ 9.58 (t, *J* = 1.5 Hz, 1H, C*H*O), 7.84 (dd, *J* = 3.0, 5.6 Hz, 2H), 7.71 (dd, *J* = 3.0, 5.6 Hz, 2H), 3.71 (t, *J* = 7.1, 2H), 2.50 (dt, *J* = 1.5, 7.1, 2H), 1.63–1.77 (m, 4H).

6-(1,3-dioxoisoindolin-2-yl)hexanal **3** was prepared from 6-amino-1-hexanol (1.011 g, 8.63 mmol) according to the procedure for **2** to yield the title compound (87 mg, 42%) as a colourless oil: *R_f_* 0.29 (EtOAc:PE, 3:7). ^1^H NMR (400 MHz, CDCl_3_) δ 9.74 (t, *J* = 1.7 Hz, 1H, C*H*O), 7.80–7.85 (m, 2H, Ar*H*), 7.68–7.73 (m, 2H, Ar*H*), 3.68 (t, *J* = 7.6 Hz, 2H, H-6), 2.43 (dt, *J* = 1.7, 7.6 Hz, 2H, H-2), 1.68 (sext, *J* = 7.6 Hz, 4H), 1.33–1.41 (m, 2H).

3-(2-methyl-1,3-dioxolan-2-yl)propanal **30** and 4-(2-methyl-1,3-dioxolan-2-yl)butanal **31** were prepared from ethyl levulinate and ethyl 4-acetylbutyrate as described by Shindo et al. [66].

#### 2.3.3. General Procedure for Cyclobutene Synthesis

##### Methyl 3-(2-(1,3-dioxoisoindolin-2-yl)ethyl)cyclobut-1-enecarboxylate **4**

To a stirred solution of **1** (1.078 g, 4.97 mmol) in MeCN (25 mL) was added diethylamine (1.03 mL, 727 mg, 9.94 mmol) and K_2_CO_3_ (1.37 g, 9.94 mmol) and the resulting suspension stirred at room temperature for 2 h. Methyl acrylate (1.13 mL, 855 mg, 9.94 mmol) was added and the reaction mixture stirred at room temperature for a further 44 h. The reaction mixture was filtered through Celite™ and the filtrate concentrated in vacuo. The residue was redissolved in MeCN (28 mL), methyl iodide (1.55 mL, 3.53 g, 24.85 mmol) added, and the resulting solution stirred at room temperature for 2 h. The volatiles were removed in vacuo, and the residue redissolved in DCE (28 mL), DBU (743 μL, 757 mg, 4.97 mmol) added, and the resulting solution heated to 75 °C for 3 h. The reaction mixture was concentrated in vacuo and the residue purified by flash column chromatography (EtOAc:PE, 3:7) to yield the title compound (851 mg, 60% over 3 steps) as a pale yellow oil: *R_f_* 0.29 (EtOAc:PE, 3:7). ^1^H NMR (400 MHz, CDCl_3_) δ 7.83–7.86 (m, 2H, Ar*H*), 7.71–7.73 (m, 2H, Ar*H*), 6.84 (d, *J* = 1.3 Hz, 1H, H-2), 3.75 (t, *J* = 6.9 Hz, 2H, C*H*_2_N), 3.72 (s, 3H, OC*H*_3_), 2.86 (dd, *J* = 4.3, 13.5 Hz, 1H, H-4), 2.72–2.78 (m, 1H, H-3), 2.32 (dd, *J* = 1.8, 13.5 Hz, 1H, H-4), 1.84–1.94 (m, 2H, C*H*_2_). ^13^C NMR (100 MHz, CDCl_3_) δ 168.3 (C), 162.9 (C), 148.9 (CH), 137.5 (C), 134.0 (CH), 132.1 (C), 123.4 (CH), 51.4 (CH_3_), 37.6 (CH), 36.5 (CH_2_), 34.8 (CH_2_), 32.0 (CH_2_). MS (ES+) *m/z* 308 ([*M*+Na]^+^, 45%), 256 ([M-OMe+H]^+^, 100). HRMS Found 286.1075, C_16_H_16_O_4_N req. 286.1074.

Methyl 3-(3-(1,3-dioxoisoindolin-2-yl)propyl)cyclobut-1-enecarboxylate **5:** white solid. mp 74–75 °C. *R_f_* 0.46 (EtOAc:PE, 3:7). ^1^H NMR (400 MHz, CDCl_3_) δ 7.79–7.84 (m, 2H, Ar*H*), 7.67–7.72 (m, 2H, Ar*H*), 6.80 (d, *J* = 1.0 Hz, 1H, H-2), 3.69 (s, 3H, OC*H*_3_), 3.68 (t, *J* = 7.2 Hz, 2H, C*H*_2_N), 2.80 (dd, *J* = 4.3, 13.3 Hz, 1H, H-4), 2.70–2.75 (m, 1H, H-3), 2.23 (dd, *J* = 1.5, 13.3 Hz, 1H, H-4), 1.65–1.75 (m, 2H, C*H*_2_CH_2_N), 1.49–1.55 (m, 2H, C*H*_2_CH_2_CH_2_N). ^13^C NMR (100 MHz, CDCl_3_) δ 168.4 (C), 163.0 (C), 149.6 (CH), 137.2 (C), 134.0 (CH), 132.1 (C), 123.2 (CH), 51.3 (CH_3_), 39.4 (CH), 37.8 (CH_2_), 34.7 (CH_2_), 30.4 (CH_2_), 26.8 (CH_2_). MS (ES+) *m/z* 322 ([*M*+Na]^+^, 100%), 300 ([*M*+H]^+^, 42%). HRMS Found 300.1228, C_17_H_18_O_4_N_1_ req. 300.1230.

Methyl 3-(4-(1,3-dioxoisoindolin-2-yl)butyl)cyclobut-1-enecarboxylate **6**: yellow oil. *R_f_* 0.40 (EtOAc:PE, 3:7). ^1^H NMR (400 MHz, CDCl_3_) δ 7.81–7.86 (m, 2H, Ar*H*), 7.68–7.73 (m, 2H, Ar*H*), 6.83 (d, *J* = 1.0 Hz, 1H, H-2), 3.71 (s, 3H, OC*H*_3_), 3.68 (dt, 2H, *J* = 2.3, 7.3 Hz, C*H*_2_N), 2.80 (dd, *J* = 4.3, 13.4 Hz, 1H, H-4), 2.66–2.71 (m, 1H, H-3), 2.23 (dd, *J* = 1.5, 13.3 Hz, 1H, H-4), 1.64–1.73 (m, 2H), 1.50–1.57 (m, 2H), 1.34–1.42 (m, 2H); ^13^C NMR (100 MHz, CDCl_3_) δ 168.4 (C), 163.1 (C), 150.1 (CH), 137.1 (C), 133.9 (CH), 132.1 (C), 123.2 (CH), 51.3 (CH_3_), 39.9 (CH), 37.8 (CH_2_), 34.8 (CH_2_), 32.8 (CH_2_), 28.6 (CH_2_), 25.1 (CH_2_). MS (ES+) *m/z* 314 ([*M*+H^+^], 100%). HRMS Found 314.1388, C_18_H_20_O_4_N_1_ req. 314.1387.

Methyl 3-((2-methyl-1,3-dioxolan-2-yl)methyl)cyclobut-1-enecarboxylate **32:** pale yellow oil. *R_f_* 0.44 (EtOAc:PE, 3:7). ^1^H NMR (400 MHz, CDCl_3_) δ 6.85 (s, 1H, H-2), 3.89–3.98 m, (4H, OC*H*_2_C*H*_2_O), 3.72 (s, 3H, OC*H*_3_), 2.89 (app qd, *J* = 2.5, 9.1 Hz, 2H, H-3, H-4), 2.33–2.37 (m, 1H, H-4), 1.87 (dd, *J* = 7.1, 14.1 Hz, 1H, C*H*H′), 1.84 (dd, *J* = 7.5, 14.1 Hz, 1H, CH*H*′), 1.33 (s, 3H, C*H*_3_). ^13^C NMR (100 MHz, CDCl_3_) δ 163.0 (C), 150.6 (CH), 136.7 (C), 109.7 (C), 64.7 (CH_2_), 51.3 (CH_3_), 42.1 (CH_2_), 35.7 (CH/CH_3_), 35.5 (CH_2_), 24.2 (CH/CH_3_). MS (AP+) *m/z* 213 ([*M*+H^+^], 100%). HRMS Found 235.0941, C_11_H_16_O_4_Na req. 235.0941.

Methyl 3-(2-(2-methyl-1,3-dioxolan-2-yl)ethyl)cyclobut-1-enecarboxylate **33:** pale yellow oil. *R_f_* 0.44 (EtOAc:PE, 3:7). ^1^H NMR (400 MHz, CDCl_3_) δ 6.84 (d, *J* = 1.0 Hz, 1H, H-2), 3.89–3.97 (m, 4H, OC*H*_2_C*H*_2_O), 3.73 (s, 3H, OC*H*_3_), 2.82 (dd, *J* = 4.0, 13.3 Hz, 1H, H-4), 2.69–2.74 (m, 1H, H-3), 2.25 (dd, *J* = 1.5, 13.3 Hz, 1H, H-4′), 1.65–1.72 (m, 2H), 1.56–1.64 (m, 2H), 1.31 (s, 3H, C*H*_3_). ^13^C NMR (100 MHz, CDCl_3_) δ 163.1 (C), 149.9 (CH), 137.2 (C), 109.8 (C), 64.7 (CH_2_), 51.2 (CH/CH_3_), 39.9 (CH/CH_3_), 37.1 (CH_2_), 34.7 (CH_2_), 27.6 (CH_2_), 23.8 (CH/CH_3_). MS (AP+) *m/z* 226.8 ([*M*+H]^+^, 100%); HRMS Found 227.1278, C_12_H_19_O_4_ req. 227.1278.

#### 2.3.4. General Procedure for Synthesis of *Cis*-Cyclobutanes

##### (1*s*,3*s*)-methyl 3-(2-(1,3-dioxoisoindolin-2-yl)ethyl)cyclobutanecarboxylate **7**

A solution of **4** (953 mg, 3.34 mmol) in ethyl acetate (40 mL) was filtered through a 1 cm silica plug. To the filtrate was added 10% Pd/C (95 mg) and the resulting suspension stirred at room temperature under 1 atm H_2_ for 22.5 h. The reaction mixture was filtered through Celite™ and concentrated in vacuo to yield the title compound (949 mg, 99%) as a pale yellow oil: *R_f_* 0.29 (EtOAc:PE, 3:7). ^1^H NMR (400 MHz, CDCl_3_) δ 7.69–7.71 (m, 2H, Ar*H*), 7.81–7.84 (m, 2H, Ar*H*), 3.64 (s, 3H, OC*H*_3_), 3.61 (t, *J* = 6.9 Hz, 2H, C*H*_2_NPth), 2.96 (tt, *J* = 8.3, 9.6 Hz, 1H, H-1), 2.28–2.36 (m, 2H, H-2,4), 2.18–2.26 (m, 1H, H-3), 1.89–1.96 (m, 2H, H-2,4), 1.78 (q, *J* = 6.9 Hz, 2H, C*H*_2_CH_2_NPth). ^13^C NMR (100 MHz, CDCl_3_) δ 175.4 (C), 168.4 (C), 134.0 (CH), 132.1 (C), 123.3 (CH), 51.6 (CH_3_), 37.1 (CH_2_), 35.2 (CH_2_), 34.5 (CH), 31.3 (CH_2_), 29.4 (CH). MS (ES+) *m/z* 326 ([*M*+K^+^], 100%).

(1*r*,3*s*)-methyl 3-(3-(1,3-dioxoisoindolin-2-yl)propyl)cyclobutanecarboxylate **8:** white solid. mp 34–35 °C. ^1^H NMR (400 MHz, CDCl_3_) δ 7.81–7.85 (m, 2H), 7.69–7.73 (m, 2H), 3.65 (t, *J* = 7.3 Hz, 2H, C*H*_2_N), 3.64 (s, 3H, OC*H*_3_), 2.95 (tt, *J* = 8.6, 9.1 Hz, 1H, H-1), 2.18–2.32 (m, 3H, H-2,4, H-3), 1.86 (dq, *J* = 2.0, 9.6 Hz, 2H, H-2,4), 1.54–1.61 (m, 2H), 1.40–1.46 (m, 2H). ^13^C NMR (100 MHz, CDCl_3_) δ 175.7 (C), 168.5 (C), 133.9 (CH), 132.1 (C), 123.2 (CH), 51.6 (CH_3_), 37.9 (CH_2_), 34.2 (CH), 33.7 (CH_2_), 31.4 (CH_2_), 31.2 (CH), 25.6 (CH_2_). MS (ES+) *m/z* 319 (]*M*+H_2_O]^+^, 78%), 302 ([*M*+H]^+^, 100). HRMS Found [*M*+NH_4_]^+^ 319.1657, C_17_H_23_O_4_N_2_ req. 319.1652.

3-[4-(1,3-Dioxo-1,3-dihydro-isoindol-2-yl)-butyl]-cyclobutanecarboxylic acid methyl ester **9:** white solid. *R_f_* 0.40 (EtOAc:PE, 3:7). mp 67–68 °C. ^1^H NMR (400 MHz, CDCl_3_) δ 7.81–7.85 (m, 2H, Ar*H*), 7.68–7.72 (m, 2H, Ar*H*), 3.64 (s, 3H, OC*H*_3_), 3.64 (t, *J* = 6.1 Hz, 2H, C*H*_2_NPth), 2.93 (tt, *J* = 8.3, 9.6 Hz, 1H, H-1), 2.23–2.30 (m, 2H, H-2,4), 2.09–2.22 (m, 1H, H-3), 1.79–1.87 m, (2H, H-2,4), 1.59–1.67 (m, 2H, C*H*_2_CH_2_NPth), 1.39–1.45 (m, 2H, C*H*_2_CH_2_CH_2_CH_2_NPth), 1.19–1.27 (m, 2H, C*H*_2_CH_2_CH_2_NPth). ^13^C NMR (100 MHz, CDCl_3_) δ 175.8 (C), 168.5 (C), 133.9 (CH), 132.1 (C), 123.2 (CH), 51.6 (CH_3_), 38.0 (CH_2_), 36.2 (CH_2_), 34.3 (CH), 31.6 (CH), 31.5 (CH_2_), 28.5 (CH_2_), 24.2 (CH_2_). MS (ES+) *m/z* 354 (100%), 316 ([*M*+H^+^], 48).

(1*s*,3*s*)-Methyl 3-((2-methyl-1,3-dioxolan-2-yl)methyl)cyclobutanecarboxylate **34:** colourless liquid. *R_f_* 0.44 (EtOAc:PE, 3:7). ^1^H NMR (400 MHz, CDCl_3_) δ 3.86–3.96 (m, 4H, OC*H*_2_C*H*_2_O), 3.65 (s, 3H, OC*H*_3_), 2.97 (tt, *J* = 7.6, 9.6 Hz, 1H, H-1), 2.29–2.42 (m, H-2,4, 3H, H-3), 1.96 (dq, *J* = 2.0, 9.6 Hz, 2H, H-2,4), 1.76 (d, *J* = 6.6 Hz, 2H, C*H*_2_), 1.26 (s, 3H, C*H*_3_). ^13^C NMR (100 MHz, CDCl_3_) δ 175.9 (C), 109.8 (C), 64.6 (CH_2_), 51.6 (CH_3_), 45.5 (CH_2_), 35.2 (CH/CH_3_), 31.3 (CH_2_), 27.6 (CH/CH_3_), 24.1 (CH/CH_3_). MS (AP+) *m/z* 215 ([*M*+H^+^], 100%). HRMS Found 215.1279, C_11_H_19_O_4_ req. 215.1278.

(1*r*,3*s*)-Methyl 3-(2-(2-methyl-1,3-dioxolan-2-yl)ethyl)cyclobutanecarboxylate **35:** grey oil. *R_f_* 0.44 (EtOAc:PE, 3:7). ^1^H NMR (400 MHz, CDCl_3_) δ 3.87–3.96 (m, 4H, OC*H*_2_C*H*_2_O), 3.65 (s, 3H, OC*H*_3_), 2.95 (tt, *J* = 8.6, 9.6 Hz, 1H, H-1), 2.29 (dq, *J* = 2.0, 8,6 Hz, 2H, H-2,4), 2.12–2.22 (m, 1H, H-3), 1.86 (dq, *J* = 2.5, 9.6 Hz, 2H, H-2,4), 1.44–1.54 (m, 4H, C*H*_2_C*H*_2_), 1.29 (m, 3H, C*H*_3_). ^13^C NMR (100 MHz, CDCl_3_) δ 175.6 (C), 109.9 (C), 64.6 (CH_2_), 51.5 (CH_3_), 36.3 (CH_2_), 34.2 (CH/CH_3_), 31.7 (CH/CH_3_), 31.4 (CH_2_), 31.0 (CH_2_), 23.7 (CH/CH_3_). MS (AP+) *m/z* 229 ([*M*+H]^+^, 100%). HRMS Found 229.1436, C_12_H_21_O_4_ req. 229.1434.

#### 2.3.5. General Procedure for the Synthesis of *Trans*-Cyclobutanes

##### 3-[3-(1,3-Dioxo-1,3-dihydro-isoindol-2-yl)-propyl]-cyclobutanecarboxylic Acid Methyl Ester **11**

To a stirred solution of **5** (500 mg, 1.67 mmol) in acetone (6 mL), water (4 mL), and conc. HCl (10 mL) was added Zn (326 mg, 5.02 mmol) and the reaction mixture heated to reflux. Four further portions of Zn (326 mg, 5.02 mmol) were added at hourly intervals. One hour after the final addition (total reaction time 6 h), the reaction mixture was cooled to room temperature, diluted with water (50 mL), and extracted with EtOAc (6 × 20 mL). The combined organic layers were dried (MgSO_4_), filtered, and concentrated in vacuo to yield crude 3-[3-(1-oxo-1,3-dihydro-isoindol-2-yl)-propyl]-cyclobutanecarboxylic acid as a yellow oil: ^1^H NMR (400 MHz, CDCl_3_) δ 7.87 (d, *J* = 7.6 Hz, 1H), 7.53 (dt, *J* = 1.5, 7.6 Hz, 1H), 7.45 (brt, *J* = 7.6 Hz, 2H), 4.38 (brs, 2H), 3.62 (brt, *J* = 7.1 Hz, 2H, C*H*_2_NH), 3.06–3.13 (m, 1H, H-1), 2.36–2.43 (m, 3H), 1.84–1.93 (m, 3H), 1.56–1.63 (m, 2H), 1.47–1.52 (m, 2H); MS (ES+) *m/z* 274 ([*M*+H^+^], 100%). To a stirred solution of this crude product in acetone (39 mL) was added Jones’ reagent (2.78 mL of a 2.7 M solution, 7.52 mmol) and the resulting solution stirred at room temperature for 18.33 h. The reaction was quenched by the dropwise addition of IPA and the resulting solution filtered through Celite, washing with EtOAc, and the filtrate concentrated in vacuo to yield crude 3-[3-(1,3-dioxo-1,3-dihydro-isoindol-2-yl)-propyl]-cyclobutanecarboxylic acid: *R_f_* 0.08 (EtOAc:PE, 3:7). mp 101–103 °C (from CDCl_3_). ^1^H NMR (400 MHz, CDCl_3_) δ 7.81–7.86 (m, 2H, Ar*H*), 7.68–7.73 (m, 2H, Ar*H*), 3.67 (t, *J* = 7.1 Hz, 2H, C*H*_2_N), 3.05–3.12 (m, 1H, H-1), 2.35–2.43 (m, 3H), 1.87–1.95 (m, 2H), 1.54–1.64 (m, 2H), 1.46–1.51 (m, 2H); ^13^C NMR (100 MHz, CDCl_3_) δ 181.6 (C), 168.5 (C), 133.9 (CH), 132.1 (C), 123.2 (CH), 38.0 (CH_2_), 34.7 (CH), 33.5 (CH_2_), 31.8 (CH), 30.2 (CH_2_), 26.2 (CH_2_). MS (ES+) *m/z* 310 ([*M*+Na]^+^, 100%). HRMS Found (*M*+NH_4_^+^) 305.1497, C_16_H_21_O_4_N_2_ req. 305.1496. To a stirred solution of this crude product in methanol (25 mL) was added SOCl_2_ (131 μL, 218 mg, 1.84 mmol) and the resulting solution heated to reflux for 27.5 h. The reaction mixture was concentrated in vacuo and the residue purified by flash column chromatography (EtOAc:PE, 3:7) to yield the title compound (343 mg, 68%) as a colourless oil: *R_f_* 0.42 (EtOAc:PE, 3:7). ^1^H NMR (400 MHz, CDCl_3_) δ 7.82–7.85 (m, 2H, Ar*H*), 7.69–7.72 m, (2H, Ar*H*), 3.67 (s, 3H, OC*H*_3_), 3.64–3.68 m, (2H, C*H*_2_N), 3.00–3.11 (m, 1H, H-1), 2.32–2.43 (m, 3H, H-2,4, H-3), 1.84–1.89 (m, 2H, H-2,4), 1.55–1.63 (m, 2H), 1.45–1.53 (m, 2H); ^13^C NMR (100 MHz, CDCl_3_) δ 176.7 (C), 168.4 (C), 133.9 (CH), 132.1 (C), 123.2 (CH), 51.7 (CH_3_), 38.0 (CH_2_), 34.7 (CH), 33.5 (CH_2_), 31.8 (CH), 30.2 (CH_2_), 26.2 (CH_2_). MS (ES+) *m/z* 324 ([*M*+Na]^+^, 100%). Found (*M*+NH_4_^+^) 319.1654, C_17_H_23_O_4_N_2_ req. 319.1652.

3-[2-(1,3-Dioxo-1,3-dihydro-isoindol-2-yl)-ethyl]-cyclobutanecarboxylic acid methyl ester **10**: colourless oil. *R_f_* 0.37 (EtOAc:PE, 3:7). ^1^H NMR (400 MHz, CDCl_3_) δ 7.81–7.84 (m, 2H), 7.69–7.71 (m, 2H), 3.66 (s, 3H, OC*H*_3_), 3.61 (t, *J* = 7.6 Hz, 2H), 3.05–3.14 (s, 1H), 2.35–2.50 (m, 3H), 1.90–1.98 (m, 2H), 1.75–1.86 (m, 2H). ^13^C NMR (100 MHz, CDCl_3_) δ 176.4 (C), 168.4 (C), 134.1 (CH), 132.1 (C), 123.4 (CH), 51.9 (CH_3_), 36.5 (CH_2_), 35.8 (CH_2_), 35.7 (CH), 30.0 (CH_2_), 29.7 (CH). MS (ES+) *m/z* 305 ([*M*+H_2_O]^+^,100%), 288 ([*M*+H^+^], 65). HRMS Found 288.1232, C_16_H_18_O_4_N req. 288.1230.

##### (1*s*,3*r*)-Methyl 3-(3-oxobutyl)cyclobutanecarboxylate **36**

To a stirred solution of **33** (308 mg, 1.36 mmol) in acetone (5.5 mL), water (3.7 mL), and concentrated HCl (8.9 mL) was added Zn (354 mg, 5.45 mmol) and the reaction mixture heated to reflux. Five further portions of Zn (5 × 354 mg, 5 × 5.45 mmol) were added at 2 hourly intervals, then the reaction mixture heated to reflux for 14 h, diluted with water (20 mL), and extracted with EtOAc (3 × 10 mL). The combined organic layers were dried (MgSO_4_), filtered, and concentrated in vacuo. The residue was redissolved in methanol (24 mL), thionyl chloride (106 μL, 178 mg, 1.50 mmol) added, and the reaction mixture heated to reflux for 24 h. The solvent was removed in vacuo and the residue purified by flash column chromatography (EtOAc:PE, 1:4) to yield the title compound (192 mg, 76%) as a brown oil: *R_f_* 0.41 (EtOAc:PE, 3:7). ^1^H NMR (400 MHz, CDCl_3_) δ 3.68 (s, 3H, OC*H*_3_), 3.03–3.11 (m, 1H, H-1), 2.26–2.42 (m, 5H, H-2,4, H-3, COC*H*_2_), 2.14 (s, 3H, C*H*_3_CO), 1.84–1.91 (m, 2H, H-2,4), 1.72 (q, *J* = 7.6 Hz, 2H, COCH_2_C*H*_2_). ^13^C NMR (100 MHz, CDCl_3_) δ 208.8 (C), 51.7 (CH/CH_3_), 41.3 (CH_2_), 34.6 (CH/CH_3_), 31.6 (CH/CH_3_), 30.1 (3CH_2_), 30.0 (CH/CH_3_). MS (ES+) *m/z* 202 ([*M*+H_2_O]^+^, 100%). HRMS Found [*M*+NH_4_]^+^ 202.1438, C_18_H_20_O_3_N req. 202.1438.

#### 2.3.6. General Procedure for Pyrimidine Incorporation

##### (1*r*,3*s*)-Methyl 3-(3-(pyrimidin-2-ylamino)propyl)cyclobutanecarboxylate **13**

To a stirred solution of 8 (51 mg, 0.169 mmol) in MeOH (15 mL) was added methylamine (650 μL of a 40% aqueous solution, 7.8 mmol) and the reaction mixture stirred at room temperature for 2 h. The reaction mixture was concentrated in vacuo and azeotroped with toluene (10 mL). The crude residue was dissolved in *n*BuOH (2 mL). 2-Chloropyrimidine (40 mg, 0.254 mmol) and DIPEA (44 μL, 33 mg, 0.254 mmol) were added and the resulting solution heated to 100 °C for 21 h. The reaction mixture was concentrated in vacuo and purified by flash column chromatography (EtOAc:PE, 3:7→1:0) to yield the title compound (25 mg, 59%) as a colourless oil: *R_f_* 0.11 (EtOAc:PE, 3:7). ^1^H NMR (400 MHz, CDCl_3_) δ 8.26 (d, *J* = 4.5 Hz, 2H, H-4′, 6′), 6.50 (t, *J* = 4.5 Hz, 1H, H-5′), 5.09 (brs, 1H, N*H*), 3.65 (s, 3H, OC*H*_3_), 3.37 (appq, *J* = 7.0 Hz, 2H, C*H*_2_NH), 2.96 (appdqn, *J* = 1.5, 8.1 Hz, 1H, H-1), 2.26–2.34 (m, 2H, H-2,4), 2.14–2.26 (m, 1H, H-3), 1.87 (appdq, *J* = 2.5, 9.6 Hz, 2H, H-2,4), 1.45–1.56 (m, 4H, C*H*_2_C*H*_2_). ^13^C NMR (100 MHz, CDCl_3_) δ 175.7 (C), 162.4 (C), 158.0 (CH), 110.4 (CH), 51.6 (CH_3_), 41.3 (CH_2_), 34.2 (CH), 33.9 (CH_2_), 31.5 (CH_2_), 31.4 (CH), 36.9 (CH_2_). MS (ES+) *m/z* 250 ([*M*+H]^+^, 100%). HRMS Found 250.1553, C_13_H_20_O_3_N_2_ req. 250.1550.

(1*s*,3*s*)-methyl 3-(2-(pyrimidin-2-ylamino)ethyl)cyclobutanecarboxylate **12:** pale yellow oil. *R_f_* 0.11 (EtOAc:PE, 3:7). ^1^H NMR (400 MHz, CDCl_3_) δ 8.26 (d, *J* = 4.8 Hz, 2H, H-4′, 6′), 6.52 (t, *J* = 4.8 Hz, 1H, H-5′), 5.42 (brs, 1H, N*H*), 3.65 (s, 3H, OC*H*_3_), 3.34 (~q, *J* = 5.8 Hz, 2H, C*H*_2_NH), 2.94–3.02 (m, 1H, H-1), 2.27–2.37 (m, 3H, H-2,4, H-3), 1.89–1.99 (m, 2H, H-2,4), 1.72 (q, *J* = 6.8 Hz, 2H, C*H*_2_CH_2_NH). ^13^C NMR (100 MHz, CDCl_3_) δ 175.5 (C), 161.8 (C), 157.8 (CH), 110.3 (CH), 51.6 (CH_3_), 39.3 (CH_2_), 36.4 (CH_2_), 34.5 (CH), 31.4 (CH_2_), 29.5 (CH). MS (ES+) *m/z* 236 ([*M*+H^+^], 100%).

3-[4-(Pyrimidin-2-ylamino)-butyl]-cyclobutanecarboxylic acid methyl ester **14:** white solid. *R_f_* 0.17 (EtOAc:PE, 3:7). ^1^H NMR (400 MHz, CDCl_3_) δ 8.26 (d, *J* = 4.8 Hz, 2H, H-4′,6′), 6.50 (t, *J* = 4.8 Hz, 1H, H-5′), 5.18 (br, 1H, N*H*), 3.65 (s, 3H, OC*H*_3_), 3.37 (dt, *J* = 6.0, 7.0 Hz, 2H, C*H*_2_N), 2.94 (tt, *J* = 8.3, 9.3 Hz, 1H, H-1), 2.23–2.31 (m, 2H, H-2,4), 2.11–2.23 (m, 1H, H-3), 1.80–1.88 (m, 2H, H-2,4), 1.57 (qn, *J* = 7.3 Hz, 2H, C*H*_2_CH_2_N), 1.39–1,45 (m, 2H, CH-3C*H*_2_), 1.23–1.32 m, (2H, C*H*_2_CH_2_CH_2_N). ^13^C NMR (100 MHz, CDCl_3_) δ 175.8 (C), 162.4 (C), 158.0 (CH), 110.4 (CH), 51.6 (CH_3_), 41.4 (CH_2_), 36.4 (CH_2_), 35.0 (CH), 31.7 (CH), 31.6 (CH_2_), 29.5 (CH_2_), 24.3 (CH_2_). MS (ES+) *m/z* 264 ([*M*+H]^+^, 100%). Found 264.1706, C_14_H_22_O_2_N_3_ req. 264.1707.

3-[2-(Pyrimidin-2-ylamino)-ethyl]-cyclobutanecarboxylic acid methyl ester **15:** pale yellow oil. *R_f_* 0.07 (EtOAc:PE, 3:7). ^1^H NMR (400 MHz, CDCl_3_) δ 8.25 (d, *J* = 4.5 Hz, 2H, H-4′,6′), 6.50 (t, *J* = 4.5 Hz, 1H, H-6′), 5.27 (brm, 1H, N*H*), 3.67 (s, 3H, OC*H*_3_), 3.33 (td, *J* = 6.0, 7.1 Hz, 2H, C*H*_2_N), 3.06–3.14 (m, 1H, H-1), 2.45–2.53 (m, 1H, H-3), 2.36–2.43 (m, 2H, H-2,4), 1.91–1.98 (m, 2H, H-2,4), 1.76 (q, *J* = 7.1 Hz, 2H, C*H*_2_CH_2_NH). ^13^C NMR (100 MHz, CDCl_3_) δ 176.5 (C), 162.4 (C), 158.0 (CH), 110.4 (CH), 51.7 (CH_3_), 39.4 (CH_2_), 36.0 (CH_2_), 34.9 (CH), 31.5 (CH_2_), 29.9 (CH). MS (ES+) *m/z* 236 ([*M*+H^+^],100%). HRMS Found 236.1396, C_12_H_18_O_2_N_3_ req. 236.1394.

(1*s*,3*r*)-Methyl 3-(3-(pyrimidin-2-ylamino)propyl)cyclobutanecarboxylate **16:** colourless oil. *R_f_* 0.10 (EtOAc:PE, 3:7). ^1^H NMR (400 MHz, CDCl_3_) δ 8.26 (d, *J* = 5.1 Hz, 2H, H-4′,6′), 6.51 (t, *J* = 5.1 Hz, 1H, H-5′), 5.08 (br, 1H, N*H*), 3.68 (s, 3H, OC*H*_3_), 3.36–3.42 (m, 2H, C*H*_2_NH), 3.04–3.10 (m, 1H, H-1), 2.33–2.44 (m, 3H, H-2,4, H-3), 1–83-1.92 (m, 2H, H-2,4), 1.52–1.55 (m, 4H, C*H*_2_C*H*_2_). ^13^C NMR (100 MHz, CDCl_3_) δ 176.7 (C), 162.4 (C), 158.1 (CH), 110.5 (CH), 51.7 (CH_3_), 41.4 (CH_2_), 34.8 (CH), 33.6 (CH_2_), 31.9 (CH), 30.3 (CH_2_), 27.2 (CH_2_). MS (ES+) *m/z* 250 ([*M*+H^+^],100%). Found 250.1550, C_13_H_20_O_3_N_2_ req. 250.1550.

#### 2.3.7. General Procedure for Friedlander Synthesis

##### (1*r*,3*s*)-Methyl 3-(2-(1,8-naphthyridin-2-yl)ethyl)cyclobutanecarboxylate **38**

To a stirred solution of **35** (1.26 g, 5.61 mmol) in methanol (50 mL) was added 5% aqueous HCl (10 mL) and the reaction mixture stirred at room temperature for 1.3 h. The volatiles were removed in vacuo and the residue purified by flash column chromatography (EtOAc:PE, 1:4) to yield (1*r*,3*s*)-methyl 3-(3-oxobutyl)cyclobutanecarboxylate (950 mg, 93%) as a pale yellow liquid: *R_f_* 0.38 (EtOAc:PE, 3:7). ^1^H NMR (400 MHz, CDCl_3_) δ 3.66 (s, 3H, OC*H*_3_), 2.95 (tt, *J* = 8.6, 9.6 Hz, 1H, H-1), 2.32 (t, *J* = 7.6 Hz, 2H, COC*H*_2_), 2.29 (dq, *J* = 2.0, 8.6 Hz, 2H, H-2,4), 2.15–2.23 (m, 1H, H-3), 2.12 (s, 3H, C*H*_3_CO), 1.87 (dq, *J* = 2.5, 9.6 Hz, 2H, H-2,4), 1.67 (q, *J* = 7.6 Hz, 2H, COCH_2_C*H*_2_). ^13^C NMR (100 MHz, CDCl_3_) δ 208.6 (C), 175.5 (C), 51.6 (CH_3_), 40.9 (CH_2_), 34.1 (CH/CH_3_), 31.2 (CH_2_), 31.0 (CH/CH_3_), 30.5 (CH_2_), 29.8 (CH/CH_3_). MS (AP+) *m/z* 185 ([*M*+H]^+^, 100%). HRMS Found [*M*+NH_4_]^+^ 202.1437, C_10_H_20_O_3_N req. 202.1438. To a stirred solution of (1*r*,3*s*)-methyl 3-(3-oxobutyl)cyclobutanecarboxylate (118 mg, 0.641 mmol) in methanol (11.8 mL) was added 2-aminonicotinaldehyde (87 mg, 0.705 mmol), pyrrolidine (59 μL, 50 mg, 0.705 mmol), and conc. H_2_SO_4_ (1 drop), and the resulting solution stirred at room temperature for 24 h. The reaction mixture was concentrated in vacuo and purified by flash column chromatography (EtOAc) to yield the title compound (178 mg, 100%) as a pale yellow oil: *R_f_* 0.05 (EtOAc:PE, 1:1). ^1^H NMR (400 MHz, CDCl_3_) δ 9.06 (dd, *J* = 1.5, 5.1 Hz, 1H, H-7′), 8.13 (dd, *J* = 2.0, 8.0 Hz, 1H, H-6′), 8.07 (d, *J* = 8.1 Hz, 1H, H-4′), 7.42 (dd, *J* = 4.0, 8.1 Hz, 1H, H-5′), 7.35 (d, *J* = 8.6 Hz, 1H, H-3′), 3.64 (s, 3H, OC*H*_3_), 2.89–2.98 (m, 3H, H-2,4, H-1), 2.27–2.34 (m, 3H, H-2,4, H-3), 1.96–2.02 (m, 2H), 1.89–1.96 (m, 2H). ^13^C NMR (100 MHz, CDCl_3_) δ 175.6 (C), 166.4 (C), 156.0 (C), 153.3 (CH), 136.9 (CH), 136.6 (CH), 122.4 (CH), 121.4 (CH), 121.0 (C), 51.5 (CH_3_), 36.6 (CH_2_), 36.1 (CH_2_), 34.2 (CH), 31.5 (CH_2_), 31.4 (CH). MS (AP+) *m/z* 271 ([*M*+H]^+^, 100%); HRMS Found 271.1444, C_16_H_19_O_2_N_2_ req. 271.1441.

(1*s*,3*s*)-Methyl 3-((1,8-naphthyridin-2-yl)methyl)cyclobutanecarboxylate **37:** pale yellow oil. *R_f_* 0.06 (EtOAc:PE, 1:1). ^1^H NMR (400 MHz, CDCl_3_) δ 9.07 (dd, *J* = 2.0, 4.5 Hz, 1H, H-7′), 8.14 (dd, *J* = 2.0, 8.1 Hz, 1H, H-5′), 8.08 (d, *J* = 8.1 Hz, 1H, H-4′), 7.43 (dd, *J* = 4.0, 8.1 Hz, 1H, H-6′), 7.33 (d, *J* = 8.1 Hz, 1H, H-3′), 3.66 (s, 3H, OC*H*_3_), 3.15 (d, *J* = 7.6 Hz, 2H, ArC*H*_2_), 3.00 (tt, *J* = 8.6, 9.6 Hz, 1H, H-1), 2.94 (ttt, *J* = 7.6, 8.1, 9.6 Hz, 1H,), 2.37 (mq, *J* = 8.1 Hz, 2H, H-2,4), 2.12 (dq, *J* = 2.5, 9.6 Hz, 2H, H-2,4). ^13^C NMR (100 MHz, CDCl_3_) δ 175.6 (C), 164.7 (C), 156.1 (C), 153.4 (CH), 137.0 (CH), 136.7 (CH), 122.7 (CH), 121.5 (CH), 121.1 (C), 51.7 (CH_3_), 45.8 (CH_2_), 34.5 (CH), 31.6 (CH_2_), 31.4 (CH). MS (AP+) *m/z* 257 ([*M*+H^+^], 100%). HRMS Found 257.1289, C_15_H_17_O_2_N_2_ req. 257.1285.

(1*s*,3*r*)-methyl 3-(2-(1,8-naphthyridin-2-yl)ethyl)cyclobutanecarboxylate **39**: yellow oil. *R_f_* 0.03 (EtOAc:PE, 1:1). ^1^H NMR (400 MHz, CDCl_3_) δ 9.06 (br, 1H), 8.14 (dd, *J* = 2.0, 8.1 Hz, 1H), 8.08 (d, *J* = 8.6 Hz, 1H), 7.42 (dd, *J* = 4.5, 8.1 Hz, 1H), 7.35 (d, *J* = 8.1 Hz, 1H), 3.66 (s, 3H, OC*H*_3_), 3.05–3.14 (m, 1H, H-1), 2.94–2.97 (m, 2H, C*H*_2_Napth), 2.35–2.52 (m, 3H, H-2,4, H-3), 2.05 (td, *J* = 7.5, 9.6 Hz, 2H, NapthCH_2_C*H*_2_), 1.91–1.98 (m, 2H, H-2,4). ^13^C NMR (100 MHz, CDCl_3_) δ 176.7 (C), 166.4 (C), 156.0 (C), 153.2 (CH), 137.0 (CH), 136.8 (CH), 122.6 (CH), 121.5 (CH), 121.0 (C), 52.1 (CH_3_), 36.9 (CH_2_), 35.9 (CH_2_), 34.8 (CH), 33.1 (CH), 32.0 (CH_2_). MS (AP+) *m/z* 271 ([*M*+H]^+^, 100%); HRMS Found 271.1445, C_16_H_19_O_2_N_2_ req. 271.1441.

#### 2.3.8. Aspartate Mimetic Synthesis

(*R*) and (*S*)-3-Amino-2-benzenesulfonylamino-propionic acid were prepared as described by Egbertson et al. [67]. (*S*)-3-amino-2-(2,4,6-trimethylphenylsulfonamido)propanoic acid was prepared as described by Pitts et al. [68].

#### 2.3.9. General Procedure for Esterification

##### 3-Amino-2-benzenesulfonylamino-propionic Acid Methyl Ester **18**, **19**

To a stirred solution of 3-amino-2-benzenesulfonylamino-propionic acid (918 mg, 3.76 mmol) in methanol (20 mL) was added thionyl chloride (300 μL, 492 mg, 4.13 mmol) and the reaction mixture stirred at room temperature for 23.5 h. The solvent was removed in vacuo and the residue dissolved in saturated aqueous NaHCO_3_ solution (40 mL) and extracted with EtOAc (8 × 15 mL). The combined organic layers were dried (MgSO_4_) and concentrated in vacuo to yield the title compound (324 mg, 33%) as a colourless oil: *R_f_* 0.10 (DCM:MeOH, 95:5): ^1^H NMR (400 MHz, CDCl_3_) δ 7.84 (d, *J* = 8.6 Hz, 2H,), 7.57 (tt, *J* = 2.0, 7.1 Hz, 1H,), 7.50 (dt, *J* = 1.5. 8.1 Hz, 2H,), 3.92 (t, *J* = 4.7, 1H,), 3.52 (s, 3H), 3.02 (dd, *J* = 4.5, 13.6 Hz, 1H), 2.98 (dd, *J* = 5.1, 13.1 Hz, 1H), Lit [69].

(*S*)-methyl 3-amino-2-(2,4,6-trimethylphenylsulfonamido)propanoate **20:** pale yellow oil. *R_f_* 0.13 (DCM:MeOH, 95:5). ^1^H NMR (400 MHz, CDCl_3_) δ 6.94 (s, 2H, ArH), 3.83 (t, *J* = 4.5 Hz, 1H, H-2), 3.56 (s, 3H, OC*H*_3_), 3.00 (dd, *J* = 4.5, 13.1 Hz, 1H, H-3), 2.98 (dd, *J* = 4.5, 13.1 Hz, 1H, H-3), 2.64 (s, 6H, ArC*H*_3_), 2.28 (s, 3H, ArC*H*_3_), Lit [70].

#### 2.3.10. General Procedure for Coupling Reactions

##### (*S*)-Methyl 3-((1*s*,3*r*)-3-(2-(pyrimidin-2-ylamino)ethyl)cyclobutanecarboxamido)-2-(2,4,6-trimethylphenylsulfonamido)propanoate **21**

Compound **12** (27 mg, 0.115 mmol) was dissolved in 6M HCl and stirred at room temperature overnight. The solvent was removed in vacuo and the residue dissolved in DMF (4 mL). (*S*)-methyl 3-amino-2-trimethylphenylsulfonamidopropanoate (34.5 mg, 0.115 mmol), EDCI hydrochloride (66 mg, 0.345 mmol), HOBt (47 mg, 0.345 mmol), and DIPEA (100 μL, 74 mg, 0.575 mmol) were added sequentially and the reaction mixture stirred at room temperature overnight. The reaction mixture was diluted with water (10 mL) and extracted with EtOAc (3 × 5 mL). The combined organic layers were washed with water (2 × 10 mL), concentrated in vacuo, and purified by PTLC (DCM:MeOH, 97:3) to yield the title compound (20 mg, 34.5%) as a colourless oil: *R_f_* 0.37 (DCM:MeOH, 95:5). [α] ${}_{D}^{20}$ + 11.5 (*c* 1.0, CHCl_3_). ^1^H NMR (400 MHz, CDCl_3_) δ 8.26 (d, *J* = 4.5 Hz, 2H, H-4′,6′), 6.94 (s, 2H, Ar*H*), 6.50 (t, *J* = 4.5 Hz, 1H, H-5′), 5.88–5.93 (m, 2H, 2N*H*), 5.14 (vbrt, 1H, N*H*Ar), 3.86 (dt, *J* = 4.0, 6.6 Hz, 1H, C*H*CHH’), 3.58–3.65 (m, 1H, C*H*H’N), 3.58 (s, 3H, OC*H*_3_), 3.48–3.56 (m, 1H, CH*H*’N), 3.32 (appq, *J* = 7.1 Hz, 2H, C*H*_2_NHAr), 2.76–2.84 (m, 1H, H-1), 2.62 (s, 6H, ArC*H*_3_), 2.25–2.31 (m + s, 6H, ArC*H*_3_, H-3, H-2,4), 1.86–1.96 (m, 2H, H-2,4), 1.71 (q, *J* = 6.6 Hz, 2H, C*H*_2_CH_2_NHAr). ^13^C NMR (100 MHz, CDCl_3_) δ 175.3 (C), 170.3 (C), 162.3 (C), 158.1 (CH), 142.7 (C), 139.2 (C), 133.0 (C), 132.1 (CH), 110.4 (CH), 55.3 (CH/CH_3_), 53.1 (CH/CH_3_), 41.8 (CH_2_), 39.3 (CH_2_), 36.3 (CH), 36.2 (CH_2_), 31.5 (CH_2_), 31.3 (CH_2_), 29.3 (CH/CH_3_), 22.9 (CH/CH_3_), 21.0 (CH/CH_3_). MS (ES+) *m/z* 504 ([*M*+H]^+^, 100%). HRMS Found 504.2275, C_24_H_34_O_5_N_5_S req. 504.2275.

3-({3-[3-(Pyrimidin-2-ylamino)-propyl]-cyclobutanecarbonyl}-amino)-propionic acid methyl ester **22:** white crystals. mp 94–95 °C. *R_f_* 0.35 (DCM:MeOH, 95:5). ^1^H NMR (400 MHz, CDCl_3_) δ 8.24 (d, *J* = 5.1, 2H, H-4′,6′), 6.49 (t, *J* = 5.1 Hz, 1H, H-4′), 5.95 (vbrt, 1H, N*H*β-ala), 5.13 (br, 1H, ArN*H*), 3.69 (s, 3H, OC*H*_3_), 3.49 (q, *J* = 6.1 Hz, 2H, C*H*_2_CH_2_CO_2_Me), 3.35 (q, *J* = 7.1 Hz, 2H, ArNHC*H*_2_), 2.74 (td, *J* = 8.1, 9.6 Hz, 1H, H-1), 2.52 (t, *J* = 6.1 Hz, 2H, C*H*_2_CO_2_Me), 2.14–2.27 (m, 3H, H-2,4, H-3), 2.14–2.27 (m, 2H, H-2,4), 1.43–1.55 (m, 4H, CH_2_CH_2_). ^13^C NMR (100 MHz, CDCl_3_) δ 174.6 (C), 173.3 (C), 162.4 (C), 158.0 (CH), 110.4 (CH), 51.8 (CH_3_), 41.3 (CH_2_), 36.2 (CH), 34.7 (CH_2_), 33.8 (CH_2_), 33.6 (CH_2_), 3.14 (CH_2_), 31.1 (CH), 27.0 (CH_2_). MS (ES+) *m/z* 321 ([*M*+H]^+^, 100%). HRMS Found 321.1926, C_16_N_25_O_3_N_4_ req. 321.1921.

(*S*)-methyl 2-(phenylsulfonamido)-3-((1*r*,3*r*)-3-(3-(pyrimidin-2-ylamino)propyl)cyclobutanecarboxamido)propanoate **23**: pale yellow oil. *R_f_* 0.05 (DCM:MeOH, 95:5). [α] ${}_{D}^{20}$ + 32.9 (*c* 0.65, CHCl_3_). ^1^H NMR (400 MHz, CDCl_3_) δ 8.26 (d, *J* = 4.6 Hz, H-4′,6′2H,), 7.82 (d, *J* = 7.1 Hz, 2H, Ar*H*), 7.54 (t, *J* = 7.6 Hz, 1H, Ar*H*), 7.50 (t, *J* = 7.6 Hz, 2H, Ar*H*), 6.49 (t, *J* = 4.5 Hz, 1H, H-5′), 6.31 (brs, 1H, N*H*SO_2_Ph), 5.97 (t, *J* = 6.1 Hz, 1H, N*H*CO), 5.30 (brm, 1H, N*H*Ar), 3.99 (brt, *J* = 5.1 Hz, 1H, C*H*NHSO_2_Ph), 3.55 (s, 3H, OC*H*_3_), 2.51–3.58 (m, 2H, C*HH*’CH), 3.36 (q, *J* = 6.1 Hz, 2H, C*H*_2_NHAr), 2.78 (qn, *J* = 8.6 Hz, 1H, H-1), 2.14–2.28 (m, 3H, H-2,4, H-3), 1.76–1.86 (m, 2H, H-2,4), 1.47–1.53 (m, 4H, C*H*_2_C*H*_2_CH_2_NHAr). ^13^C NMR (100 MHz, CDCl_3_) δ 176.7 (C), 170.1 (C), 162.0 (C), 158.0 (CH), 139.3 (C), 133.1 (CH), 129.2 (CH), 127.1 (CH), 110.3 (CH), 55.7 (CH/CH_3_), 53.0 (CH/CH_3_), 41.7 (CH_2_), 41.3 (CH_2_), 36.0 (CH/CH_3_), 33.7 (CH_2_), 31.4 (CH_2_), 31.2 (CH/CH_3_), 26.8 (CH_2_). MS (ES+) *m/z* 476 ([*M*+H]^+^, 100%). HRMS Found 476.1953, C_22_H_30_O_5_N_5_S req. 476.1962.

(*S*)-methyl 3-((1*r*,3*r*)-3-(pyrimidin-2-ylamino)propyl)cyclobutane-1-carboxamido)-2-((2,4,6-trimethylphenyl)sulfonamido)propanoate **24**: pale yellow oil. *R_f_* 0.27 (DCM:MeOH, 95:5); [α] ${}_{D}^{20}$ + 7.8 (*c* 0.6, CHCl_3_). ^1^H NMR (400 MHz, CDCl_3_) δ 8.26 (d, *J* = 5.1 Hz, 2H, H-4′,6′), 6.94 (s, 2H, Ar*H*), 6.50 (t, *J* = 5.1 Hz, 1H, H-5′), 6.06 (brd, *J* = 7.1 Hz, 1H, N*H*SO_2_Ar), 5.90 (brt, *J* = 5.7 Hz, 1H, N*H*CO), 5.20 (brt, *J* = 5.6 Hz, 1H, N*H*Ar), 3.83–3.88 (m, 1H, C*H*CHH’), 3.58 (s, 3H, OC*H*_3_), 3.45–3.56 (m, 2H, CHC*HH*’), 3.34–3.40 (m, 2H, C*H*_2_NHAr), 2.78 (qn, *J* = 8.6 Hz, 1H, H-1), 2.62 (s, 6H, ArC*H*_3_), 21.6–2.30 (m + s, ArC*H*_3_, 6H, H-3, H-2,4), 1.77–1.87 (m, 2H, H-2,4), 1.46–1.55 (m, 4H, C*H*_2_C*H*_2_CH_2_NHAr). ^13^C NMR (100 MHz, CDCl_3_) δ 175.5 (C), 170.3 (C), 162.4 (C), 158.1 (CH), 146.8 (C), 142.7 (C), 139.2 (C), 132.1 (CH), 110.4 (CH), 55.2 (CH/CH_3_), 53.1 (CH/CH_3_), 41.7 (CH_2_), 41.3 (CH_2_), 36.1 (CH/CH_3_), 33.7 (CH_2_), 31.4 (CH/CH_3_), 31.3 (CH_2_), 26.9 (CH_2_), 22.9 (CH/CH_3_), 21.0 (CH/CH_3_). MS (ES+) *m/z* 518 ([*M*+H]^+^, 100%). HRMS Found 518.2431, C_25_H_36_O_5_N_5_S_1_ req. 518.2432.

(*R*)-methyl 2-(phenylsulfonamido)-3-((1*r*,3*s*)-3-(3-(pyrimidin-2-ylamino)propyl)cyclobutanecarboxamido)propanoate **25**: pale yellow oil. *R_f_* 0.05 (DCM:MeOH, 95:5); [α] ${}_{D}^{20}$ − 26.99 (*c* 0.715, CHCl_3_). ^1^H NMR (400 MHz, CDCl_3_) δ 8.26 (d, *J* = 4.6 Hz, 2H, H-4′,6′), 7.82 (d, *J* = 7.1 Hz, 2H, Ar*H*), 7.54 (t, *J* = 7.6 Hz, 1H, Ar*H*), 7.50 (t, *J* = 7.6 Hz, 2H, Ar*H*), 6.49 (t, *J* = 4.5 Hz, 1H, H-5′), 6.31 (brs, 1H, N*H*SO_2_Ph), 5.97 (t, *J* = 6.1 Hz, 1H, N*H*CO), 5.30 (brm, 1H, N*H*Ar), 3.99 (brt, *J* = 5.1 Hz, 1H, C*H*NHSO_2_Ph), 3.55 (s, 3H, OC*H*_3_), 2.51–3.58 (m, 2H, C*HH*’CH), 3.36 (q, *J* = 6.1 Hz, 2H, C*H*_2_NHAr), 2.78 (qn, *J* = 8.6 Hz, 1H, H-1), 2.14–2.28 (m, 3H, H-2,4, H-3), 1.76–1.86 (m, 2H, H-2,4), 1.47–1.53 (m, 4H, C*H*_2_C*H*_2_CH_2_NHAr). ^13^C NMR (100 MHz, CDCl_3_) δ 176.7 (C), 170.1 (C), 162.0 (C), 158.0 (CH), 139.3 (C), 133.1 (CH), 129.2 (CH), 127.1 (CH), 110.3 (CH), 55.7 (CH/CH_3_), 53.0 (CH/CH_3_), 41.7 (CH_2_), 41.3 (CH_2_), 36.0 (CH/CH_3_), 33.7 (CH_2_), 31.4 (CH_2_), 31.2 (CH/CH_3_), 26.8 (CH_2_). MS (ES+) *m/z* 498 ([M+Na]^+^, 63%), 476 ([M+H]^+^, 100). HRMS Found [M+H^+^] 476.1966, C_22_H_30_N_5_O_5_S req. 476.1962.

(*S*)-methyl 3-((1*r*,3*r*)-3-(4-(pyrimidin-2-ylamino)butyl)cyclobutanecarboxamido)-2-(2,4,6-trimethylphenylsulfonamido)propanoate **26**: pale yellow oil. *R_f_* 0.18 (DCM:MeOH, 95:5). [α] ${}_{D}^{20}$ + 25.2 (*c* 1.05, CHCl_3_). ^1^H NMR (400 MHz, CDCl_3_) δ 8.26 (d, *J* = 5.1 Hz, 2H, H-4′,6′), 6.94 (s, 2H, Ar*H*), 6.50 (t, *J* = 5.1 Hz, 1H, H-5′), 5.96 (brd, *J* = 7.6 Hz, 1H, N*H*SO_2_Ar), 5.89 (brt, *J* = 5.7 Hz, 1H, N*H*CO), 5.19 (vbrt, *J* = 5.5 Hz, 1H, N*H*Ar), 3.86 (dt, *J* = 4.0, 7.6 Hz, 1H, C*H*NHSO_2_Ar), 3.57 (s, 3H, CO_2_C*H*_3_), 3.47–3.66 (m, 2H, C*H*_2_NHCO), 3.37 (q, *J* = 7.1 Hz, 2H, C*H*_2_NHAr), 2.70 (tt, *J* = 8.6, 9.6 Hz, 1H, H-1), 2.61 (s, 6H, ArC*H*_3_), 2.28 (s, 3H, ArC*H*_3_), 2.11–2.28 (m, 3H, H-2,4, H-1), 1.76–1.84 (m, 2H, H-2,4), 1.57 (qn, *J* = 7.1 Hz, 2H, C*H*_2_CH_2_NHAr), 1.39–1.44 (m, 2H, C*H*_2_CH_2_CH_2_CH_2_NHAr), 1.25–1.32 (m, 2H, C*H*_2_CH_2_CH_2_NHAr). ^13^C NMR (100 MHz, CDCl_3_) δ 175.6 (C), 170.3 (C), 162.3 (C), 158.1 (CH), 142.7 (C), 139.2 (C), 134.3 (C), 132.1 (CH), 123.5 (C), 110.3 (CH), 55.3 (CH/CH_3_), 53.0 (CH/CH_3_), 41.7 (CH_2_), 41.4 (CH_2_), 36.2 (CH_2_), 36.1 (CH), 31.4 (CH_2_), 31.3 (CH), 29.4 (CH_2_), 24.2 (CH_2_), 22,9 (CH_3_), 21.0 (CH_3_). MS (ES+) *m/z* 532 ([M+H]^+^, 100%). HRMS Found 532.2592, C_26_H_38_O_5_N_5_S_1_ req. 532.2588.

(*S*)-methyl 3-((1*r*,3*s*)-3-(2-(pyrimidin-2-ylamino)ethyl)cyclobutanecarboxamido)-2-(2,4,6-trimethylphenylsulfonamido)propanoate **27**: pale yellow oil. *R_f_* 0.38 (DCM:MeOH, 95:5). [α] ${}_{D}^{20}$ + 26.4 (*c* 1.23, CHCl_3_). ^1^H NMR (400 MHz, CDCl_3_) δ 8.25 (d, *J* = 5.1 Hz, 2H, H-4′,6′), 6.94 (s, 2H, Ar*H*), 6.50 (t, *J* = 5.1 Hz, 1H, H-5′), 5.96 (brt, *J* = 6.1 Hz, 2H, 2N*H*), 5.18 (brt, *J* = 5.1 Hz, 1H, ArN*H*), 3.88 (brm, 1H, C*H*NHSO_2_Ar), 3.57 (s, 3H, OC*H*_3_), 3.52–3.65 (m, 2H, C*H*_2_NHCO), 3.33 (dt, *J* = 6.1, 7.1 Hz, 2H, C*H*_2_NH), 2.90–2.97 (m, 1H, H-1), 2.61 (s, 6H, 2ArC*H*_3_), 2.28 (s, 3H, ArC*H*_3_), 2.33–2.48 (m, 3H, H-2,4, H-3), 1.86–1.95 (m, 2H, H-2,4), 1.76 (q, *J* = 7.1 Hz, 2H, C*H*_2_CH_2_NH). ^13^C NMR (100 MHz, CDCl_3_) δ 176.2 (C), 170.3 (C), 162.4 (C), 158.0 (CH), 142.6 (C), 139.2 (C), 133.1 (C), 132.1 (CH), 110.4 (CH), 55.3 (CH/CH_3_), 53.0 (CH/CH_3_), 41.8 (CH_2_), 39.5 (CH_2_), 36.3 (CH/CH_3_), 36.0 (CH_2_), 31.3 (CH_2_), 30.9 (CH/CH_3_), 22.9 (CH/CH_3_), 20.9 (CH/CH_3_). MS (ES+) *m/z* 504 (M+H^+^, 100%). HRMS Found 504.2267, C_24_H_34_O_5_N_5_S req. 504.2275.

(*S*)-methyl 3-((1*s*,3*s*)-3-(3-(pyrimidin-2-ylamino)propyl)cyclobutanecarboxamido)-2-(2,4,6-trimethylphenylsulfonamido)propanoate **28**: pale yellow oil. *R_f_* 0.30 (DCM:MeOH, 95:5). [α] ${}_{D}^{20}$+ 34.4 (*c* 1.23, CHCl_3_). ^1^H NMR (400 MHz, CDCl_3_) δ 8.26 (d, *J* = 4.5 Hz, 2H, H-4′,6′), 6.94 (s, 2H, Ar*H*), 6.50 (t, *J* = 4.5 Hz, 1H, H-5), 6.05 (br, 1H, N*H*), 5.94 (t, *J* = 5.7 Hz, 1H, N*H*CO), 5.25 (vbrt, 1*H*, N*H*CH_2_), 3.89 (brt, 1*H*, C*H*NHSO_2_Ph), 3.57 (s, 3H, OC*H*_3_), 3.49–3.64 (m, 2H, C*H*_2_CHNHSO_2_Ph), 3.36–3.43 (m, 2H, NHC*H*_2_), 2.86–2.93 (m, 1H, H-1), 2.62 (s, 6H, ArC*H*_3_), 2.29–2.34 (m, 3H, H-2,4, H-3), 2.28 (s, 3H, ArC*H*_3_), 1.77–1.86 (m, 2H, H-2,4), 1.51–1.54 (m, 4H, NHCH_2_C*H*_2_C*H*_2_). ^13^C NMR (100 MHz, CDCl_3_) δ 176.4 (C), 170.3 (C), 162.4 (C), 158.0 (CH), 143.6 (C), 139.2 (C), 133.2 (C), 132.0 (CH), 110.4 (CH), 55.4 (CH/CH_3_), 53.0 (CH/CH_3_), 41.7 (CH_2_), 41.4 (CH_2_), 36.4 (CH/CH_3_), 34.1 (CH_2_), 31.8 (CH/CH_3_), 30.5 (CH_2_), 27.2 (CH/CH_3_), 22.9 (CH/CH_3_), 20.9 (CH/CH_3_). MS (ES+) *m/z* 518 ([M+H]^+^, 100%). HRMS Found 518.2429, C_25_H_36_O_5_N_5_S req. 518.2432.

(*S*)-methyl 2-(phenylsulfonamido)-3-((1*s*,3*s*)-3-(3-(pyrimidin-2-ylamino)propyl)cyclobutanecarboxamido)propanoate **29**: pale yellow oil. *R_f_* 0.22 (DCM:MeOH, 95:5). [α] ${}_{D}^{20}$ + 16.8 (*c* 1.4, CHCl_3_). ^1^H NMR (400 MHz, CDCl_3_) δ 8.27 (d, *J* = 5.1 Hz, 2H, H-4′,6′), 7.83 (td, *J* = 1.5, 7.5 Hz, 2H, Ar*H*-o), 7.57 (tt, *J* = 1.5, 7.5 Hz, 1H, Ar*H*-p), 7.49 (t, *J* = 7.6 Hz, 2H, Ar*H*-m), 6.50 (t, *J* = 5.1 Hz, 1H, H-5′), 6.26 (vbr, 1H, N*H*), 5.99 (brt, *J* = 6.3 Hz, 1H, N*H*CO), 5.33 (brt, *J* = 5.1 Hz, 1H, N*H*Ar), 4.02 (dd, *J* = 4.5, 6.7 Hz, 1H, CH_2_C*H*NHSO_2_Ph), 3.55 (s, 3H, OC*H*_3_), 3.52–3.61 (m, 2H, C*H*_2_CHNHSO_2_Ph), 3.38 (brq, *J* = 6.1 Hz, 2H, C*H*_2_NHAr), 2.85–2.93 (m, 1H, H-1), 2.24–2.37 (m, 3H, H-2,4, H-3), 1.78–1.85 (m, 2H, H-2,4), 1.51–1.53 (m, 4H, C*H*_2_C*H*_2_CH_2_NHAr). ^13^C NMR (100 MHz, CDCl_3_) δ 176.5 (C), 170.2 (C), 162.4 (C), 158.1 (CH), 139.4 (C), 133.2 (CH), 129.2 (CH), 127.1 (CH), 110.4 (CH), 55.8 (CH/CH_3_), 53.0 (CH/CH_3_), 41.8 (CH_2_), 41.4 (CH_2_), 36.4 (CH/CH_3_), 33.6 (CH_2_), 31.8 (CH/CH_3_), 30.4 (CH_2_), 30.3 (CH_2_), 27.1 (CH_2_). MS (ES+) *m/z* 476 ([M+H]^+^, 100%). HRMS Found 476.1960, C_22_H_30_O_5_N_5_S req. 476.1962.

(*S*)-methyl 3-((1*r*,3*r*)-3-(2-(1,8-naphthyridin-2-yl)ethyl)cyclobutanecarboxamido)-2-(phenylsulfonamido)propanoate **40**: colourless oil. [α] ${}_{D}^{20}$ + 0.057 (*c* 1.35, CHCl_3_). *R_f_* 0.15 (DCM:MeOH, 95:5). ^1^H NMR (400 MHz, CDCl_3_) δ 9.08 (dd, *J* = 2.0, 4.0 Hz, 1H) 8.16 (dd, *J* = 2.0, 8.1 Hz, 1H), 8.09 (dd, *J* = 8.1 Hz, 1H), 7.82 (dd, *J* = 1.5, 7.6 Hz, 2H, Ph*H*-o), 7.56 (tt, *J* = 1.5, 7.6 Hz, 1H, Ph*H*-p), 7.49 (tm, *J* = 7.6 Hz, 2H, Ph*H*-m), 7.44 (dd, *J* = 4.0, 8.1 Hz, 1H), 7.38 (d, *J* = 8.6 Hz, 1H), 6.13 (brt, *J* = 6.1 Hz, 1H, N*H*), 6.07 (vbr, 1H, N*H*), 4.01 (dd, *J* = 4.0, 6.1 Hz, 1H, C*H*NHSO_2_Ph), 3.57 (s, 3H, OC*H*_3_), 3.49–3.65 (m, 2H, C*HH*’NH), 2.97 (dd, *J* = 7.6, 8.1 Hz, 2H, C*H*_2_Napth), 2.70 (tt, *J* = 8.1, 9.1 Hz, 1H, H-1), 2.20–2.33 (m, 3H, H-2,4, H-3), 1.98 (brq, *J* = 7.1 Hz, 2H, H-2,4), 1.83–1.89 (m, 2H, C*H*_2_). ^13^C NMR (100 MHz, CDCl_3_) δ 175.6 (C), 170.1 (C), 166.6 (C), 155.9 (C), 153.3 (CH), 139.6 (C), 137.0 (CH), 136.8 (CH), 132.9 (CH), 129.3 (CH), 127.1 (CH), 122.6 (CH), 121.4 (CH), 121.1 (C), 55.7 (CH/CH_3_), 52.9 (CH/CH_3_), 41.6 (CH_2_), 26.6 (CH_2_), 36.0 (CH), 35.8 (CH_2_), 31.3 (CH_2_), 31.2 (CH). MS (ES+) *m/z* 497 ([M+H]^+^, 77%), 241 (55), 121 (100). HRMS Found 497.1846, C_25_H_29_O_5_N_4_S req. 497.1853.

(*S*)-methyl 3-((1*r*,3*r*)-3-(2-(1,8-naphthyridin-2-yl)ethyl)cyclobutanecarboxamido)-2-(2,4,6-trimethylphenylsulfonamido)propanoate **41**: colourless oil. [α] ${}_{D}^{20}$ + 0.24 (*c* 0.5, CHCl_3_). *R_f_* 0.39 (DCM:MeOH, 95:5). ^1^H NMR (400 MHz, CDCl_3_) δ 9.08 (dd, *J* = 2.0, 4.0 Hz, 1H), 8.15 (dd, *J* = 2.0, 8.1 Hz, 1H), 8.09 (d, *J* = 8.1, 1H), 7.44 (dd, *J* = 4.0, 8.1 Hz, 1H), 7.38 (d, *J* = 8.1 Hz, 1H), 6.93 (s, 2H), 5.98 (t, *J* = 6.1 Hz, 1H, N*H*), 5.89 (d, *J* = 7.1 Hz, 1H, N*H*), 3.89 (dt, *J* = 4.0, 7.1 Hz, 1H, C*H*NHSO_2_), 3.61 (ddd, *J* = 4.5, 6.6, 14.1 Hz, 1H, C*H*H’NH), 3.58 s, (3H, OC*H*_3_), 3.51 (td, *J* = 6.1, 14.1 Hz, 1H, CH*H*’NH), 2.97 (dd, *J* = 7.1, 8.1 Hz, 2H, ArC*H*_2_), 2.71–2.81 (m, 1H, H-1), 2.61 (s, 6H, ArC*H*_3_), 2.28 (s, 3H, ArC*H*_3_), 2.21–2.31 (m, 3H, H-2,4, H-3), 1.99 (brq, *J* = 6.6 Hz, 2H, H-2,4), 1.84–1.90 (m, 2H). ^13^C NMR (100 MHz, CDCl_3_) δ 175.5 (C), 170.3 (C), 166.5 (C), 157.6 (C), 153.3 (CH), 142.6 (C), 139.2 (C), 136.9 (CH), 136.7 (CH), 133.3 (C), 132.0 (CH), 122.5 (CH), 121.4 (CH), 121.0 (C), 55.4 (CH/CH_3_), 52.9 (CH/CH_3_), 41.7 (CH_2_), 36.7 (CH_2_), 36.1 (CH/CH_3_), 35.8 (CH_2_), 31.4 (CH_2_), 31.3 (CH_2_), 31.2 (CH/CH_3_), 22.8 (CH/CH_3_), 20.9 (CH/CH_3_). MS (ES+) *m/z* 539 ([M+H]^+^, 100%). HRMS Found 539.2316, C_28_H_35_O_5_N_4_S req. 539.2323.

Methyl 3-((1*r*,3*s*)-3-(2-(1,8-naphthyridin-2-yl)ethyl)cyclobutanecarboxamido)propanoate **42**: white crystals. mp 101–102 °C. *R_f_* 0.17 (DCM:MeOH, 95:5). ^1^H NMR (400 MHz, CDCl_3_) δ 9.06 (dd, *J* = 2.0, 4.0 Hz, 1H), 8.15 (dd, *J* = 2.0, 8.1 Hz, 1H), 8.08 (d, *J* = 8.6 Hz, 1H), 7.43 (dd, *J* = 4.5, 8.1 Hz, 1H), 7.37 (d, *J* = 8.1 Hz, 1H), 5.97 (vbrt, 1H, N*H*), 3.68 (s, 3H, OC*H*_3_), 3.49 (q, *J* = 6.1 Hz, 2H, NHC*H*_2_), 2.95 (dd, *J* = 6.1, 7.6 Hz, 2H, ArC*H*_2_), 2.71–2.79 (m, 1H, H-1), 2.52 (t, *J* = 6.1 Hz, 2H, NHCH_2_C*H*_2_), 2.21–2.29 (m, 3H, H-2,4, H-3), 1.97 (brq, *J* = 7.3 Hz, 2H, ArCH_2_C*H*_2_), 1.82–1.92 (m, 2H, H-2,4). ^13^C NMR (100 MHz, CDCl_3_) δ 174.7 (C), 173.2 (C), 166.5 (C), 155.9 (C), 153.3 (CH), 137.0 (CH), 136.8 (CH), 122.5 (CH), 121.4 (CH), 121.0 (C), 51.8 (CH_3_), 36.7 (CH2), 36.2 (CH), 36.1 (CH_2_), 34.7 (CH_2_), 33.9 (CH_2_), 31.8 (CH_2_), 31.4 (CH). MS (AP+) *m/z* 342 ([M+H]^+^, 100%). HRMS Found 364.1640 (M+Na^+^), C_19_H_23_O_3_N_3_Na req. 364.1632.

(*S*)-methyl 3-((1*s*,3*r*)-3-((1,8-naphthyridin-2-yl)methyl)cyclobutanecarboxamido)-2-(2,4,6-trimethylphenylsulfonamido)propanoate **43**: colourless oil. [α] ${}_{D}^{20}$ + 0.127 (*c* 0.55, CHCl_3_). *R_f_* 0.28 (DCM:MeOH, 95:5). ^1^H NMR (400 MHz, CDCl_3_) δ 9.08 (dd, *J* = 1.5, 4.0 Hz, 1H, H-7′), 8.16 (dd, *J* = 1.5, 8.1 Hz, 1H, H-5′), 8.05 (d, *J* = 8.6 Hz, 1H, H-4′), 7.45 (dd, *J* = 4.5, 8.1 Hz, 1H, H-6′), 7.34 (d, *J* = 8.6 Hz, 1H, H-3′), 6.91 (s, 2H, Ar*H*), 6.44 (t, *J* = 6.1 Hz, 1H, N*H*SO_2_Mes), 6.20 (br, 1H, N*H*), 3.93 (dd, *J* = 4.0, 7.1 Hz, 1H, C*H*NHSO_2_Mes), 3.65 (dd, *J* = 4.5, 6.6, 14.1 Hz, 1H, C*H*H’CH), 3.56 (s, 3H, OC*H*_3_), 3.48–3.59 (m 1H, CH*H*’CH), 3.15 (d, *J* = 7.1 Hz, 2H, NapthC*H*_2_), 2.79–3.05 (m, 2H, H-1,3), 2.60 (s, 6H, ArC*H*_3_), 2.26 (s, 3H, ArC*H*_3_), 2.26–2.39 (m, 2H, H-2,4), 2.10–2.21 (m, 2H, H-2,4). ^13^C NMR (100 MHz, CDCl_3_) δ 175.7 (C), 170.4 (C), 164.9 (C), 155.9 (C), 153.3 (CH), 142.5 (C), 139.2 (C), 136.9 (CH), 133.3 (CH), 123.0 (CH), 121.1 (CH), 55.5 (CH/CH_3_), 53.4 (CH_2_), 52.8 (CH/CH_3_), 45.0 (CH_2_), 41.6 (CH_2_), 36.4 (CH/CH_3_), 31.4 (CH_2_), 30.8 (CH/CH_3_), 23.0 (CH/CH_3_), 20.9 (CH/CH_3_). MS (AP+) *m/z* 525 ([M+H]^+^, 9%), 121 (100). HRMS Found 525.2160, C_27_H_33_O_5_N_4_S req. 525.2166.

(*S*)-methyl 3-((1*s*,3*s*)-3-(2-(1,8-naphthyridin-2-yl)ethyl)cyclobutanecarboxamido)-2-(2,4,6-trimethylphenylsulfonamido)propanoate **44**: pale brown gum. *R_f_* 0.30 (DCM:MeOH, 95:5). [α] ${}_{D}^{20}$ + 0.134 (*c* 1.55, CHCl_3_). ^1^H NMR (400 MHz, CDCl_3_) δ 9.07 (dd, *J* = 2.0, 4.0 Hz, 1H), 8.16 (dd, *J* = 2.0, 8.1 Hz, 1H), 8.09 (d, *J* = 8.6 Hz, 1H), 7.44 (dd, *J* = 4.0, 8.1 Hz, 1H), 7.37 (d, *J* = 8.1 Hz, 1H), 6.92 (s, 2H, Ar*H*), 6.16 (brt, *J* = 6.1 Hz, 1H, N*H*CH_2_), 6.07 (vbrd, *J* = 5.1 Hz, 1H, N*H*SO_2_Ar), 3.89 (br, 1H, C*H*NHSO_2_Ar), 3.56 (s, 3H, OC*H*_3_), 3.53–3.64 (m, 2H, NHC*HH*’), 2.90–2.97 (m, 3H, NapthC*H*_2_, H-1), 2.27 (s, 6H, ArC*H*_3_), 2.29–2.43 (m, 3H, H-2,4, H-3), 2.27 (s, 3H, ArC*H*_3_), 2.04 (td, *J* = 7.6, 10.6 Hz, 2H, NapthCH_2_C*H*_2_), 1.84–1.92 (m, 2H, H-2,4); ^13^C NMR (100 MHz, CDCl_3_) δ 176.4 (C), 170.4 (C), 166.5 (C), 156.0 (C), 153.5 (CH), 142.6 (C), 139.2 (C), 137.1 (CH), 136.8 (CH), 133.2 (C), 132.2 (CH), 122.7 (CH), 121.5 (CH), 121.1 (C), 55.4 (CH/CH_3_), 52.9 (CH/CH_3_), 41.7 (CH_2_), 37.0 (CH_2_), 36.3 (CH/CH_3_), 35.8 (CH_2_), 31.9 (CH/CH_3_), 30.3 (CH_2_), 30.1 (CH_2_), 22.9 (CH/CH_3_), 21.0 (CH/CH_3_). MS (ES+) *m/z* 539 ([M+H]^+^, 100%). HRMS Found 539.2318, C_28_H_35_O_5_N_4_S req. 539.2323.

#### 2.3.11. General Procedure for Tetrahydronapthyridine Synthesis

##### (S)-methyl 3-((1*s*,3*r*)-3-((5,6,7,8-tetrahydro-1,8-naphthyridin-2-yl)methyl)cyclobutanecarboxamido)-2-(2,4,6-trimethylphenylsulfonamido)propanoate **48**

To a stirred solution of **43** (24 mg, 0.046 mmol) in methanol (5 mL) was added PtO_2_ (4 mg) and the reaction mixture stirred under 1 atm H_2_ for 24 h. The reaction mixture was filtered through Celite™ and concentrated in vacuo to yield the title compound (21 mg, 87.5%) as a colourless oil: [α] ${}_{D}^{20}$ + 0.038 (*c* 1.05, MeOH). *R_f_* 0.28 (DCM:MeOH, 95:5). ^1^H NMR (400 MHz, CDCl_3_) δ 8.25 (br, 1H), 7.33 (d, *J* = 6.6 Hz, 1H), 6.92 (s, 2H), 6.37 (dd, *J* = 7.6 Hz, 1H), 6.27 (br, 1H), 3.96 (br, 1H), 3.46–3.57 (m, 5H), 3.18 (qn, *J* = 7.6 Hz, 1H), 2.52–2.95 (m, 4H), 1.23–2.35 (m, 8H), 1.85–2.05 (m, 6H), 1.40.1.47 (m, 4H). ^13^C NMR (100 MHz, CDCl_3_) δ 172.2 (C), 170.2 (C), 168.7 (C), 148.1 (C), 142.6 (C), 140.7 (CH), 139.2 (C), 131.9 (CH), 110.4 (CH), 119.2 (C), 114.7 (C), 55.4 (CH/CH_3_), 52.7 (CH/CH_3_), 41.4 (CH_2_), 41.1 (CH_2_), 39.2 (CH_2_), 35.8 (CH/CH_3_), 30.7 (CH_2_), 30.4 (CH/CH_3_), 30.4 (CH_2_), 25.5 (CH_2_), 23.0 (CH/CH_3_), 20.9 (CH/CH_3_), 19.3 (CH_2_). MS (AP+) *m/z* 529 ([*M*+H]^+^, 8%), 246 (63), 200 (100). HRMS Found 529.2473, C_27_H_37_O_5_N_4_S req. 529.2749.

(*S*)-methyl 2-(phenylsulfonamido)-3-((1*r*,3*r*)-3-(2-(5,6,7,8-tetrahydro-1,8-naphthyridin-2-yl)ethyl)cyclobutanecarboxamido)propanoate **45**: yellow oil. [α] ${}_{D}^{20}$ + 0.113 (*c* 0.15, CHCl_3_). *R_f_* 0.41 (DCM:MeOH, 95:5). ^1^H NMR (400 MHz, CDCl_3_) δ 7.85 (dd, *J* = 1.5, 8.1 Hz, 2H, ArH-o), 7.58 (tt, *J* = 1.5, 8.1 Hz, 1H, ArH-p), 7.50 (dt, *J* = 1.5, 8.1 Hz, 2H, ArH-m), 7.12 (d, *J* = 7.4 Hz, 1H, H-4′), 6.32 (d, *J* = 7.4 Hz, 1H, H-3′), 6.14 (br, 1H, N*H*), 5.89 (vbr, 1H, N*H*), 3.91 (t, *J* = 5.2, 1H, C*H*NHSO_2_Ph), 3.57–3.60 (m, 2H, C*HH*’CHNHSO_2_Ph), 3.57 (s, 3H, C*H*_3_), 3.42 (dt, *J* = 2.5, 6.0, 2H, H-7′), 2.81 (qn, *J* = 8.6, 1H, H-1), 2.70 (t, *J* = 6.1 Hz, 2H, H-5′), 2.48 (dt, *J* = 3.0, 8.6 Hz, 2H, ArC*H*_2_), 2.14–2.32 (m, 3H, H-2,4, H-3), 1.82–1.94 (m, 4H, H-2,4, H-6′), 1.77 (q, *J* = 7.5 Hz, 2H, ArCH_2_C*H*_2_). ^13^C NMR (100 MHz, CDCl_3_) δ 175.8 (C), 170.1 (C), 139.5 (C), 137.7 (C), 133.0 (CH), 129.2 (CH), 127.2 (CH), 124.1 (C), 110.8 (CH), 55.8 (CH/CH_3_), 53.0 (CH/CH_3_), 41.5 (2CH_2_), 36.6 (CH_2_), 36.1 (CH/CH_3_), 31.3 (CH_2_), 31.0 (CH_2_), 31.0 (CH/CH_3_), 26.1 (CH_2_), 20.9 (CH_2_). MS (ES+) *m/z* 502 ([M+H]^+^, 100%). HRMS Found 501.2157, C_25_H_33_O_5_N_4_S req. 501.2166.

(*S*)-methyl 3-((1*r*,3*r*)-3-(2-(5,6,7,8-tetrahydro-1,8-naphthyridin-2-yl)ethyl)cyclobutanecarboxamido)-2-(2,4,6-trimethylphenylsulfonamido)propanoate **46**: colourless oil. [α] ${}_{D}^{20}$ + 0.113 (*c* 0.3, CHCl_3_). *R_f_* 0.27 (DCM:MeOH, 95:5). ^1^H NMR (400 MHz, CDCl_3_) δ 7.12 (d, *J* = 7.3 Hz, 1H, H-4′), 6.94 (s, 2H, Ar*H*), 6.33 (d, *J* = 7.3 Hz, 1H, H-3′), 6.03 (br, 1H, N*H*), 5.88 (vbr, 2H, 2N*H*), 3.89 (dd, *J* = 4.0, 6.6 Hz, 1H, C*H*NHSO_2_), 3.57 (s, 3H, OC*H*_3_), 3.49–3.64 (m, 2H, NHC*HH*’CH), 3.41–3.44 (m, 2H, H-7′), 2.79 (qn, *J* = 8.6 Hz, 1H, H-1), 2.70 (t, *J* = 6.3 Hz, 2H, H-5′), 2.62 (s, 6H, ArC*H*_3_), 2.48 (dd, *J* = 7.6, 8.1 Hz, 2H, ArC*H*_2_CH_2_), 2.29 (s, 3H, ArC*H*_3_), 2.16–2.99 (m, 3H, H-2,4, H-3), 1.83–1.94 (m, 4H, H-6′+H-2,4), 1.77 (q, *J* = 7.6 Hz, 2H, ArCH_2_C*H*_2_). ^13^C NMR (100 MHz, CDCl_3_) δ 175.6 (C), 170.3 (C), 155.1 (C), 142.6 (C), 139.5 (C), 139.2 (C), 137.2 9 (C), 133.2 (C), 132.0 (3 CH), 111.0 (CH), 55.4 (CH/CH_3_), 52.9 (CH/CH_3_), 41.6 (CH_2_), 41.5 (CH_2_), 36.5 (CH/CH_3_), 36.1 (CH_2_), 34.5 (CH_2_), 31.3 (CH_2_), 31.2 (CH_2_), 31.0 (CH/CH_3_), 26.2 (CH_2_), 23.1 (CH/CH_3_), 21.2 (CH/CH_3_). MS (ES+) *m/z* 543 ([M+H]^+^, 100%). HRMS Found 543.2629, C_28_H_39_O_5_N_4_S req. 543.2636.

Methyl 3-((1*r*,3*s*)-3-(2-(5,6,7,8-tetrahydro-1,8-naphthyridin-2-yl)ethyl)cyclobutanecarboxamido)propanoate **47**: white crystals. mp 117–119 °C. *R_f_* 0.31 (DCM:MeOH, 95:5). ^1^H NMR (400 MHz, CDCl_3_) δ 7.05 (d, *J* = 7.1 Hz, 1H, H-4′), 6.30 (d, *J* = 7.6 Hz, 1H, H-3′), 5.96 (brt, 1H, N*H*CH_2_), 4.95 (brs, 1H, NH-8′), 3.69 (s, 3H, OC*H*_3_), 3.49 (q, *J* = 6.0 Hz, 2H, C*H*_2_NH), 3.39 (dt, *J* = 2.5, 6.1 Hz, 2H, H-7′), 2.75 (tt, *J* = 8.1, 9.6 Hz, 1H, H-1), 2.68 (t, *J* = 6.0 Hz, 2H, H-5′), 2.53 (t, *J* = 6.0 Hz, 2H, C*H*_2_CH_2_NH), 2.43 (dd, *J* = 6.1, 7.6 Hz, 2H, C*H*_2_Ar), 2.13–2.33 (m, 3H, H-2,4, H-3), 1.79–1.92 (m, H-6′, 4H, H-2,4), 1.73 (td, *J* = 7.1, 9.6 Hz, 2H, C*H*_2_CH_2_Ar). ^13^C NMR (100 MHz, CDCl_3_) δ 174.8 (C), 173.3 (C), 157.7 (C), 155.5 (C), 136.8 (CH), 113.4 (C), 111.1 (CH), 51.8 (CH_3_), 41.6 (CH_2_), 36.6 (CH_2_), 36.3 (CH), 35.0 (CH_2_), 34.7 (CH_2_), 33.9 (CH_2_), 31.4 (CH_2_), 31.1 (CH), 26.3 (CH_2_), 21.4 (CH_2_). MS (AP+) *m/z* 346 ([M+H]^+^, 100%), 239 (67). HRMS Found 346.2127, C_19_H_28_N_3_O_3_ req. 346.2125.

(*S*)-methyl 3-((1*s*,3*s*)-3-(2-(5,6,7,8-tetrahydro-1,8-naphthyridin-2-yl)ethyl)cyclobutanecarboxamido)-2-(2,4,6-trimethylphenylsulfonamido)propanoate **49**: yellow oil. *R_f_* 0.50 (DCM:MeOH, 95:5). [α] ${}_{D}^{20}$ + 0.11 (*c* 1.05, CHCl_3_). ^1^H NMR (400 MHz, CDCl_3_) δ 7.20 (d, *J* = 7.1 Hz, 1H), 6.93 (s, 2H, Ar*H*), 6.35 (m, 1H, N*H*CH), 6.32 (d, *J* = 7.1 Hz, 1H), 3.90 (dd, *J* = 4.5, 6.0 Hz, 1H, C*H*NHSO_2_Ar), 3.54 (s, 3H, OC*H*_3_), 3.51–3.60 (m, 2H, C*HH*’CH), 2.91–2.98 (m, 2H, NHC*H*_2_CH_2_CH_2_), 2.91–2.98 (m, 1H, H-1), 2.71 (brt, *J* = 6.1 Hz, 2H, NHCH_2_CH_2_C*H*_2_), 2.62 (s, 6H, ArC*H*_3_), 2.53–2.65 (m, 2H, PyrC*H*_2_), 2.29–2.39 (m, 3H, H-2,4, H-3), 2.28 (s, 3H, ArC*H*_3_), 1.82–1.92 (m, 6H, NHCH_2_C*H*_2_CH_2_ + H2,4 + PyrCH_2_C*H*_2_). ^13^C NMR (100 MHz, CDCl_3_) δ 176.5 (C), 170.3 (C), 151.7 (C), 142.5 (C), 139.2 (C), 139.0 (CH), 133.3 (C), 132.0 (CH), 127.6 (C), 122.5 (C), 110.5 (CH), 55.5 (CH/CH_3_), 52.78 (CH/CH_3_), 41.7 (CH_2_), 41.3 (CH_2_), 36.4 (CH/CH_3_), 36.0 (CH_2_), 31.6 (CH/CH_3_), 30.3 (CH_2_), 30.2 (CH_2_), 25.9 (CH_2_), 23.1 (CH/CH_3_), 21.0 (CH/CH_3_), 20.3 (CH_2_). MS (ES+) *m*/*z* 543 ([M+H]^+^, 100%). HRMS Found 543.2630, C_28_H_39_O_5_N_4_S req. 543.2636.

Compound purity was estimated from the integration of ^1^H NMR spectra: The following compounds contained no detectable organic impurities (purity > 95%): **21**, **22**, **27**, **28**, **40**, **41**, **42**, **44**, **45**, **46**, and **48**. Other compounds had the following purities: **23**, 94%; **24**, 90%; **25**, 95%; **26**, 95%; **29**, 90%; **43**, 85%; **47**, 90%; and **49** 85%. This is a limitation of the work.

#### 2.3.12. General Procedure for Preparation of Free Acids

(*S*)-2-(phenylsulfonamido)-3-((1*r*,3*r*)-3-(3-(pyrimidin-2-ylamino)propyl) cyclobutanecarboxamido)propanoic acid **51** as a yellow oil. Compound **23** was dissolved in 6 M aqueous HCl (1 mL) and stirred at room temperature for 23.5 h. The volatiles were removed in vacuo to yield the title compound which was used immediately. MS (ES+) *m/z* 479 ([*M*+H_2_O]^+^, 100%). HRMS Found 479.2141 ([*M*+H_2_O]^+^), C_21_H_29_N_5_O_6_S req. 479.1839.

(*S*)-3-((1*s*,3*r*)-3-(2-(pyrimidin-2-ylamino)ethyl)cyclobutanecarboxamido)-2-(2,4,6-trimethylphenylsulfonamido)propanoic acid **50**: yellow oil. MS (ES-) *m/z* 488 ([M-H]^−^, 100%). HRMS Found 488.1972, C_23_H_30_O_5_N_5_S req. 488.1973.

(*S*)-3-((1*r*,3*s*)-3-(2-(pyrimidin-2-ylamino)ethyl)cyclobutanecarboxamido)-2-(2,4,6-trimethylphenylsulfonamido)propanoic acid **52**: yellow oil. MS (ES+) *m/z* 507 ([M+H_2_O, 100%), 490 ([M+H^+^], 54%). HRMS found 488.1971([M-H]^-^). C_23_H_30_O_5_N_5_S req. 488.1973.

(*S*)-3-((1*s*,3*s*)-{3-[3-(Pyrimidin-2-ylamino)-propyl]-cyclobutanecarbonyl}-amino)-2-(2,4,6-trimethyl-benzenesulfonylamino)-propionic acid **53:** yellow oil. MS (ES+) *m/z* 504 ([*M*+H]^+^ 100%). HRMS Found 504.2269, C_24_H_34_N_5_O_5_S req. 504.2275.

(*S*)-2-(phenylsulfonamido)-3-((1*s*,3*s*)-3-(3-(pyrimidin-2-ylamino)propyl) cyclobutanecarboxamido)propanoic acid **54**: yellow oil. MS (ES+) m/z 479 ([M+H_2_O]^+^, 100%). HRMS Found 479.2141 ([M+H_2_O]^+^), C_21_H_29_N_5_O_6_S req. 479.1839.

(*S*)-3-{(1*r*,3*r*)-[3-(2-[1,8] Naphthyridin-2-yl-ethyl)-cyclobutanecarbonyl]-amino}-2-(2,4,6-trimethyl-benzenesulfonylamino)-propionic acid **55**: yellow oil. MS (ES+) *m/z* 525 ([*M*+H]^+^, 100%). HRMS Found 525.2163; C_27_H_33_O_5_N_4_S req. 525.2172.

(*S*)-3-((1*r*,3*r*)-3-(2-(5,6,7,8-tetrahydro-1,8-naphthyridin-2-yl)ethyl)cyclobutanecarboxamido)-2-(2,4,6-trimethylphenylsulfonamido)propanoic acid **56**: yellow oil. MS (ES+) *m/z* 529.4 ([*M*+H]^+^, 100%). Found 527.2330 C_27_H_35_O_5_N_4_S req.527.2334.

### 2.4. ELISA Assay

ELISA assays were performed as previously described [71] with minor modifications. Briefly, serial dilutions of compounds were prepared in dimethyl sulfoxide (DMSO). C8 Maxi Immuno modules (Fisher Scientific) were incubated overnight at 4 °C with 0.5 μg/well of fibrinogen (Sigma) in sterile phosphate buffered saline (PBS, 0.2 g/L KCl, 8.0 g/L NaCl, 0.2 KH_2_PO_4_, 1.15 g/L Na_2_HPO_4_, pH 7.4). All subsequent wash steps were performed using 25 mM Tris, pH 7.6, 150 mM NaCl, 1 mM MnCl_2_, 1 mg/mL BSA, and binding/inhibition was carried out in 25 mM Tris, pH 7.6, 150 mM NaCl, 1 mM MnCl_2_, 1 mM MgCl_2_, 1 mM CaCl_2_, 1 mg/mL BSA.

Wells were washed and blocked with a blocking solution (PBS, 0.1% Tween 20, 3% BSA) for 1 h at 37 °C. Compounds were added to the wells at indicated concentrations (final 0.5% DMSO) in the presence of 0.5 µg/well α_IIb_β_3_ (Sigma) and incubated at room temp for 1 h. After 3 washes, primary anti-α_IIb_ (1:200 dilution, Santa Cruz Biotech, Heidelberg, Germany) and antigoat–HRP (1:500 dilution, Dako, Agilent, Santa Clara, CA, USA) were added at room temp for 1 h. The wells were washed and incubated with 0.1 mg/mL tetra-methylbenzidine (Sigma) for 25 min and the reaction was stopped with 1N H_2_SO_4_ (100 μL/well). The absorbance was measured at 450 nm using a Multiscan Spectrum reader (Thermo Scientific, Thermofisher, UK) and SkanIT RE (v2.4.4.5) software. Results were plotted as percent binding vs. log concentration and IC_50_ values determined.

### 2.5. Cell-Binding Assay

First, 96-well plates (Corning, VWR) were coated overnight at 4 °C with 0.5 μg/well fibronectin (Sigma). SK-Mel-2 cells were trypsinised, washed and resuspended in RPMI medium only at 10^5^ cells/mL. Cells (100 µL/well) were treated with compound (DMSO final concentration 0.1%) at the indicated concentration for 4 hrs on a rotary shaker. Plates were washed (3 × PBS) and blocked with PBS/5% BSA for 2 hrs at 37 °C prior to adding the cells. Cells were incubated on the plates at 37 °C in a humidified chamber with 5% CO_2_ for 1 hr. The plates were washed 3× with PBS and a 200 µL/well RPMI medium containing 10% FCS added. The plates were incubated overnight at 37 °C as above. Finally, 0.5 mg/mL of 3-(4,5-dimethythiazol-2-yl)-2,5-diphenyl tetrazolium bromide (MTT) was added to each well and the plates incubated for a further 4 h. The medium was removed, and the insoluble formazan dissolved in 150 µL of DMSO. Absorbance was measured at 540 nm using a Thermo Multiskan EX (Thermofisher, UK)and Ascent Software (v2.6). Total binding was determined based on controls lacking any compounds (100% binding) in fibronectin-coated wells and uncoated wells blocked with BSA (0% binding) and corrected for background (no cells, fibronectin-coated).

### 2.6. Migration Assay

M14 cells (4 × 10^5^/mL in RPMI 1640 medium) were seeded in six-well plates and incubated at 37 °C in a 5% CO_2_ humidified atmosphere for 48 h. The resulting confluent monolayer was scratched with a sterile P200 pipette tip to create a gap (approximately 2 cm in length and 650 μm in width) in the centre of the well. The medium was removed, and the cells washed with Hank’s balanced salt solution (HBSS) (1 mL) and replaced with a medium containing the test antagonist. After 24 h, the cells were washed twice with HBSS and fixed with ice-pre-cooled methanol for thirty minutes at −20 °C. Following hydration with two washes in PBS, the monolayers were counterstained with Harris’s haematoxylin solution for two minutes, then washed in tap water for one minute and left to dry at room temperature. The plates were observed using an inverted microscope, and digital images captured. The scratch width was measured at ten positions throughout the scratch area and the % inhibition calculated by comparing the average migration into the scratch at 24 h to that of the untreated control. Immunolabelling for Ki-67 (AB9260, Chemicon Millipore Watford, Watford, UK) showed low levels of nuclear expression after 24 h, confirming the migration rather than proliferation.

### 2.7. Platelet Aggregation Assay

Platelet aggregation was measured in hirudin-anticoagulated whole blood from a healthy donor with ADP as agonist (end concentration 6.4 μM) using a Multiplate^®^ impedance aggregometer (Dynabyte Informationssysteme GmbH, Munich, Germany). At 37 °C, cells were charged with 300 μL of saline, 300 μL of blood, 1 μL of compound (0.1% DMSO) and incubated for 3 min. The agonist was added and the increase in electrical impedance measured from 2 electrode pairs/cell for 6 min, transformed into arbitrary units (AU), and the area under the curve was calculated.

## 3. Results

### 3.1. Design and Synthesis of Cyclobutane-Based RGD-Mimetics

**Figure 1:**
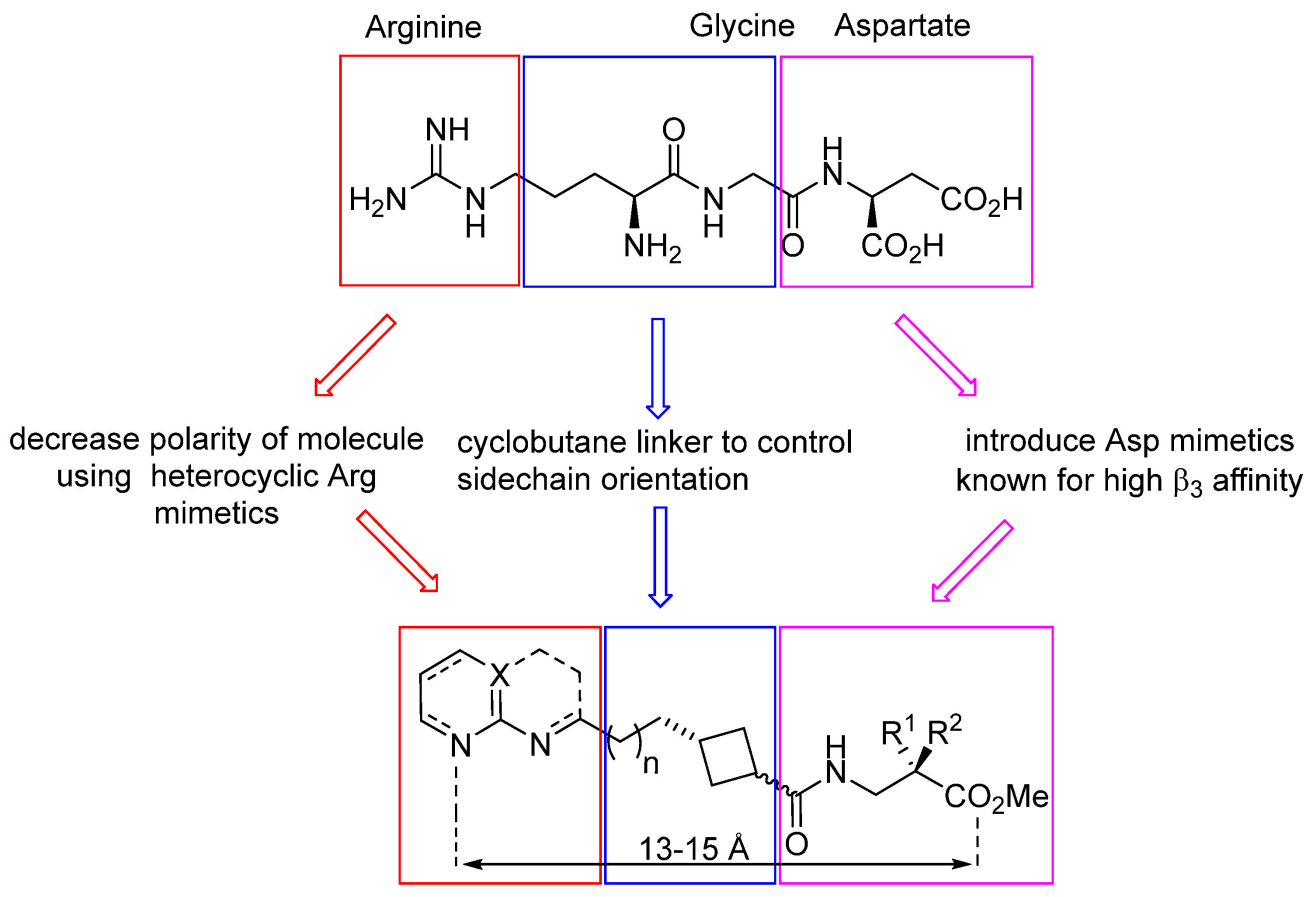
Design of RGD-mimetic integrin antagonists.

A wide range of nitrogen-containing functional groups have been used as arginine sidechain mimetics in integrin antagonists, with the aim of obtaining a high integrin affinity while improving bioavailability by reducing the basicity of the sidechain compared to guanidine. Our general approach to RGD-mimetics is outlined in Figure 1. To develop dual β3 antagonists, we selected heterocycles that would be able to achieve the dual-point binding mode required for αvβ3 binding via interaction with αv Asp218 [72]. The activity against αIIbβ3 can be obtained provided one of the sidechain nitrogen atoms is able to bind in the single point mode with αIIb via Asp224 [73], and binding to both integrins is optimised by varying the sidechain length [74,75]. NMR studies on RGD-containing peptides have indicated that the optimum separation between charged Arg and Asp groups is 14–15 Å for binding *α*IIbβ3 and ≤13 Å for *α*vβ3 [74].

**Figure 2:**
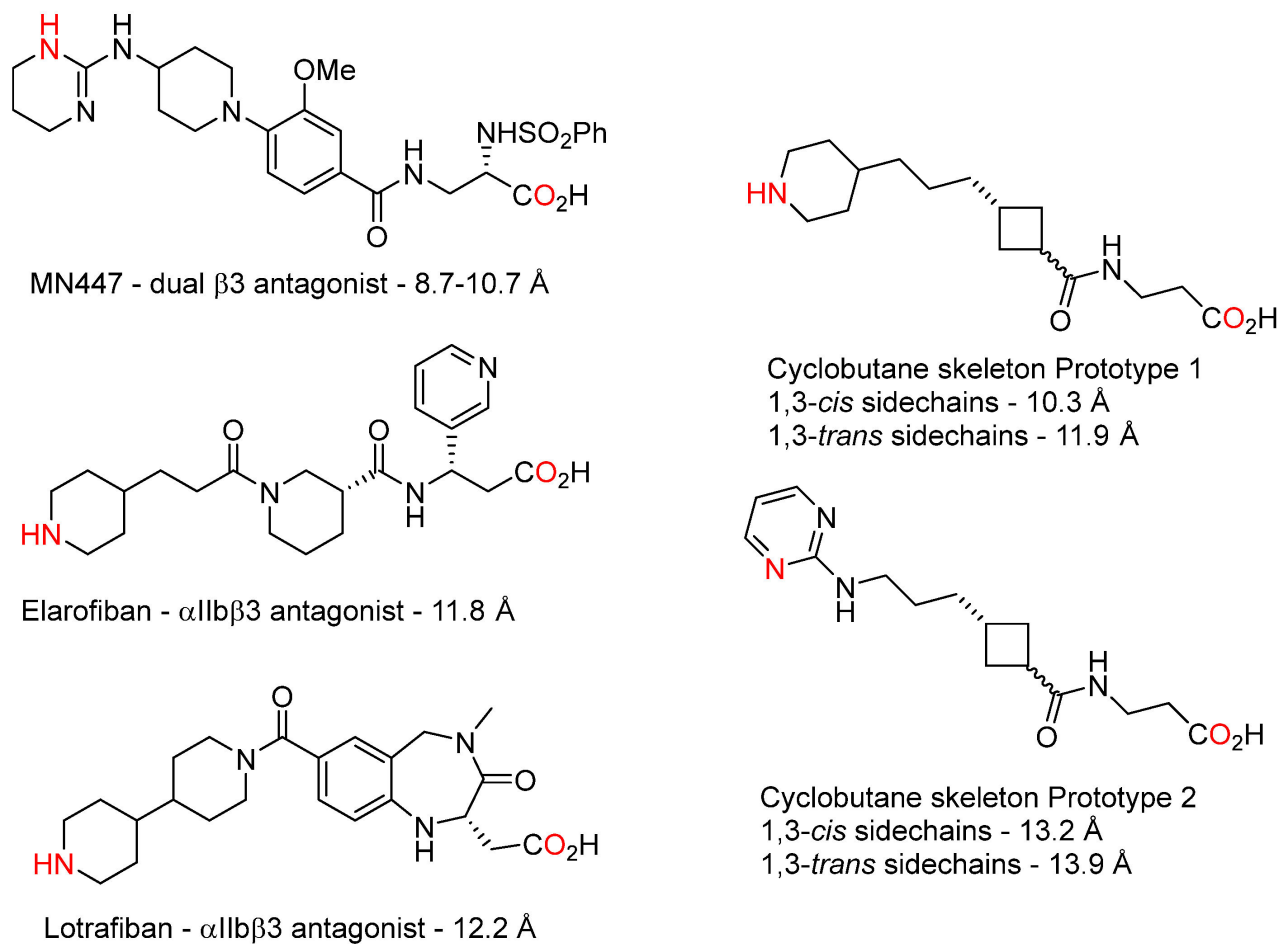
Structures modelled using Arguslab. Distances in angstroms refer to the distance between the Arg and Asp sidechain mimetic heteroatoms involved in integrin binding (indicated in red).

The feasibility of small-molecule dual β3 antagonists has been demonstrated by MN447 [50,76], which was designed to mimic the integrin binding profile of Abciximab. We used molecular modelling (Arguslab [77]) to explore the minimised molecular geometries of some known αIIbβ3-targeted small molecules and prototypical cyclobutanes (Figure 2). Interestingly, the known αIIbβ3 ligands MN447, elarofiban, and lotrafiban returned shorter lengths than anticipated. A 1,3-*cis* arrangement of cyclobutane sidechains generally gave molecules with a sidechain mimetic separation 2–3 Å shorter than the corresponding 1,3-*trans* arrangement although the difference was much less pronounced with pyrimidine-bearing sidechains. Since the cyclobutanes had low energy conformations similar to known integrin antagonists and consistent with the literature on αvβ3 and αIIbβ3 ligand length requirements, we decided to investigate the synthesis and anti-integrin properties of cyclobutanes with a 2–4 carbon sidechain bearing the Arg mimetic.

**Scheme 1:**
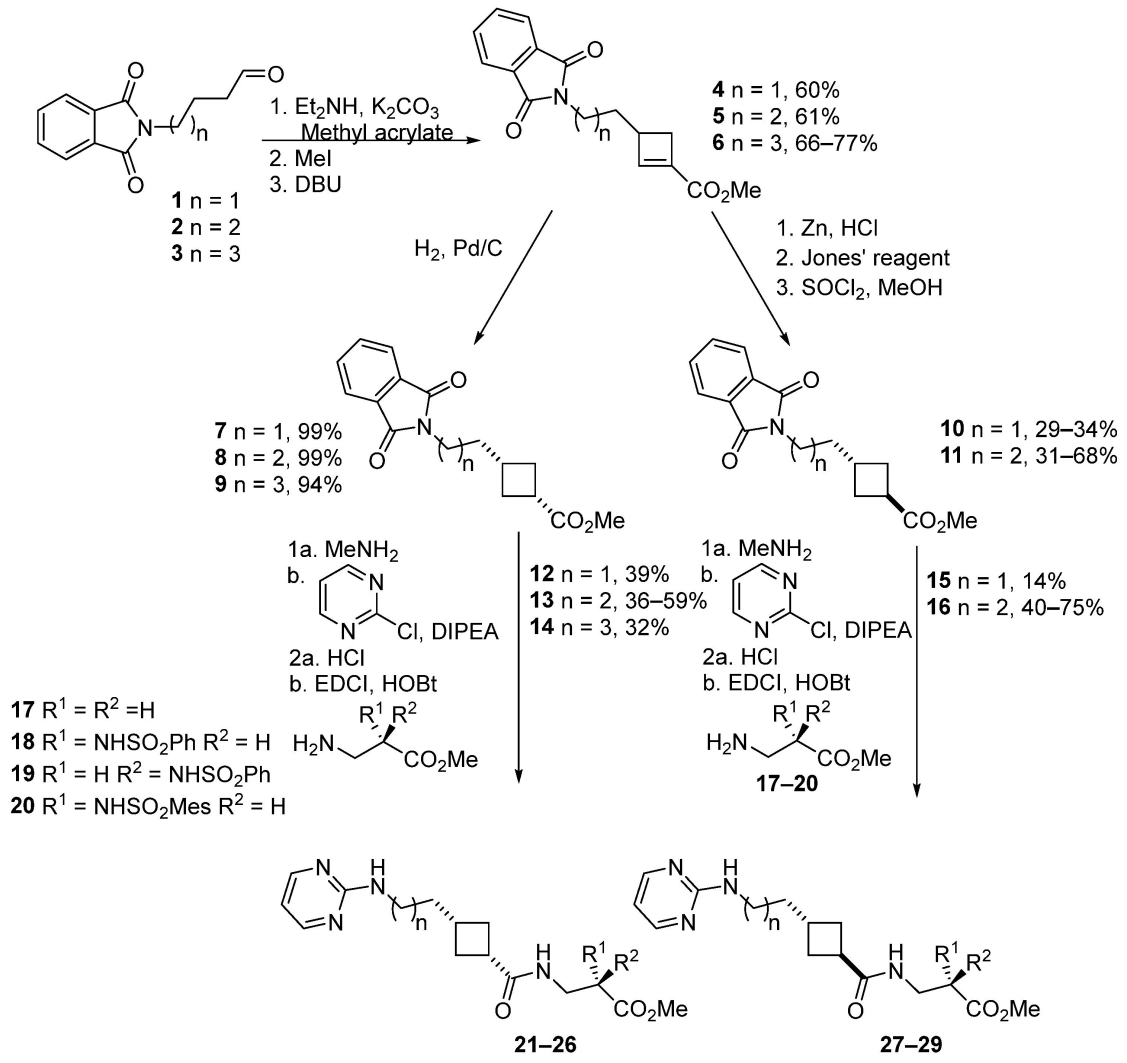
Synthesis of cyclobutanes with a pyrimidine-containing sidechain.

Guanidine analogues such as 2-aminotetrahydropyrimidines [78] and 2-aminoimidazoles [79] have previously been used in molecules with dual anti-β3 activity. Our investigation started with analogous 2-aminopyrimidines. The pyrimidine containing RGD mimetics were synthesised starting from protected aldehydes **1–3**. Double protection of the amine group was necessary for the success of the cyclobutane synthesis, and the phthalate group was found to be most suitable due to its ease of introduction and removal and compatibility with reaction conditions throughout the synthesis. Cyclobutenes **4–6** were obtained in good yield using our one-pot thermal [2+2] cyclisation methodology followed by immediate quaternisation of the resulting cyclobutylamine and Hoffmann elimination (Scheme 1) [60].

*Cis*-substituted cyclobutanes **7–9** were obtained by hydrogenation of cyclobutanes **4–6**. *Trans*-substituted cyclobutanes **10–11** were obtained from the same starting material using a modification of Dehmlow’s zinc reduction procedure [80]. The harsh reaction conditions of the zinc reduction also partially reduced the phthalate group and hydrolysed the ester; the resulting crude acids were treated with Jones reagent followed by acidic methanol to restore the original functionality. The *cis* and *trans* isomers were clearly distinguishable by their ^1^H NMR spectra and were obtained in ratios of >95:<5 *cis:trans* from the hydrogenation reaction and 1:10 *cis:trans* from the zinc reduction; in the latter case, the undesired *cis* isomer was removed by flash chromatography.

The cyclobutanes **7–11** were deprotected using methylamine [81] and the resulting crude amines heated with 2-chloropyrimidine to incorporate the pyrimidine Arg mimetic. The esters were then hydrolysed and the crude acid coupled with aspartate mimetic esters **17–20** to provide integrin antagonists **21–29**.

**Scheme 2:**
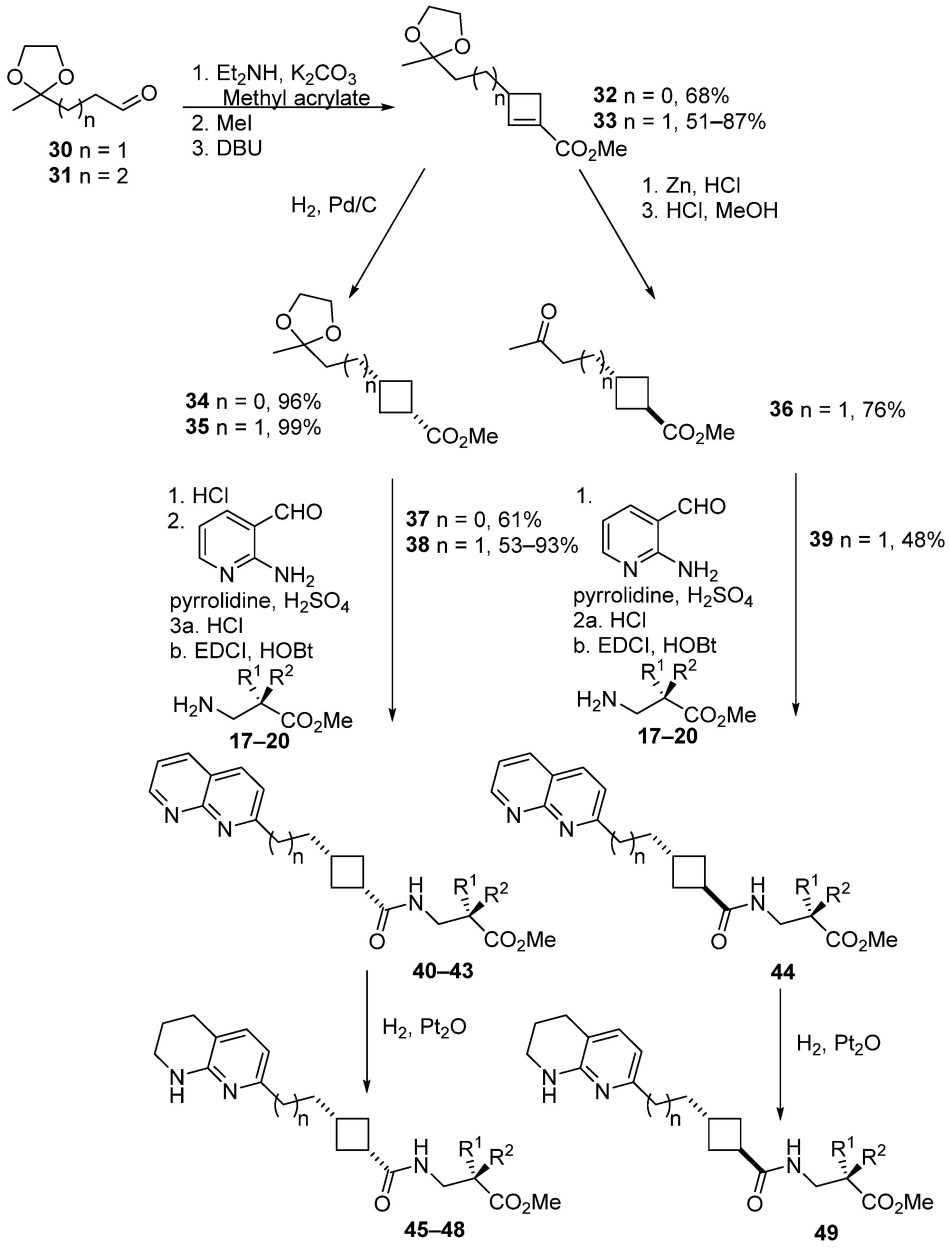
Synthesis of cyclobutanes with naphthyridine and tetrahydronaphthyridine sidechains.

Tetrahydronaphthyridines have been previously used to increase αvβ3 affinity in β3 antagonists [82]. Naphthyridine and THN-containing integrin antagonists were synthesised from protected aldehydes **30–31** (Scheme 2). Cyclobutylamine formation, quaternisation, and elimination gave the desired cyclobutenes **32–33** in good yield. The corresponding cis-cyclobutanes **34** and **35** were obtained in excellent yield by hydrogenation and the trans-cyclobutane by zinc reduction, which conveniently also removed the acetal to afford the ketone **36**. The cis-cyclobutanes were deprotected by a brief treatment with HCl followed by the formation of the naphthyridine group by a Friedlander reaction. Naphthyridines **37–39** were subjected to ester hydrolysis and coupled with Arg mimetic amines **17–20** to yield the naphthyridine series of integrin antagonists **40–44**. Hydrogenation over Pt_2_O yielded the corresponding tetrahydronaphthyridines **45–49**.

**Scheme 3:**
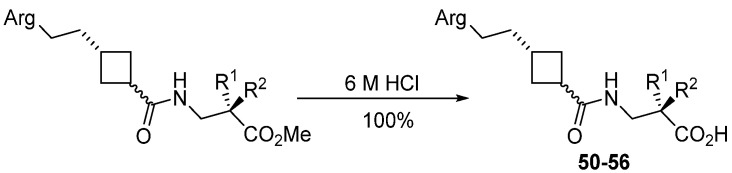
General synthesis of free acids from cyclobutyl esters.

We rationalised that the methyl esters would be rapidly hydrolysed by esterases in cells and in vivo to yield the acid group required for binding to the β3 MIDAS [72,73], and the presence of methyl ester would improve the lipid solubility and PK properties of the compounds [83,84,85]. The free acids required to test the compounds in cell-free assays were obtained from selected RGD-mimetic esters by a treatment with aqueous HCl (Scheme 3). An overall summary of the compounds synthesized is presented in Table 1.

### 3.2. Investigation of Anti-β3 Integrin Activity

As an initial test of anti-β3 activity, the ability of compounds to inhibit the adhesion of melanoma cells expressing high levels of αvβ3 and low levels of other RGD-binding integrins such as αvβ5 and α5β1 to immobilised fibronectin was evaluated. The characterisation of integrin subunit expression in Sk-Me-2 and M14 melanoma cells (Supplementary Information Figures S1–S3) confirmed previous studies showing high αv and β3 in these lines [86,87,88]. αIIb was not detectable.

Cells were seeded onto precoated plates in the presence or absence of putative antagonists and the number of adherent cells quantified colorimetrically. No binding was observed to BSA-coated plates (negative control). Linear RGDS (a nonspecific antagonist of RGD-binding integrins) and cRGDfV (a more selective αvβ3 antagonist) inhibited adhesion to the same extent, indicating cell adhesion was predominantly mediated by αvβ3 (Table 2). While determining the optimum number of cells to use for the assay, it was observed that Sk-Mel-2 cells bound effectively to fibronectin-coated surfaces with a low proportion of cells remaining unbound in the absence of compounds, but a large proportion of M14 cells did not bind despite having similar αvβ3 levels. Therefore, Sk-Mel-2 cells were used in the adhesion assay.

Before use in cell-based assays of integrin function, the effect of compounds on cell proliferation was tested using the MTT assay to avoid adhesion results being confounded by cell death (Supplementary Table S1). The effect of compounds on cell adhesion was then initially tested at a single, nontoxic concentration to identify active antagonists for further characterisation; those displaying a promising activity in the adhesion assay were tested at a wider range of concentrations to determine the IC_50_ (Table 2). This identified THN compound ICT9055 **46** (IC_50_ 0.34 μM) and its free acid ICT9064 **56** (IC_50_ 3.7 μM), and pyrimidine-free acid ICT9090 **53** (IC_50_ 11 μM) as hits of interest. It is important to note that the cell-based assay gives higher IC_50_s than observed in purified protein binding [89], and there is a higher degree of variation in results with less active compounds. Nevertheless, it is a cost-effective initial screening method.

**Figure 3:**
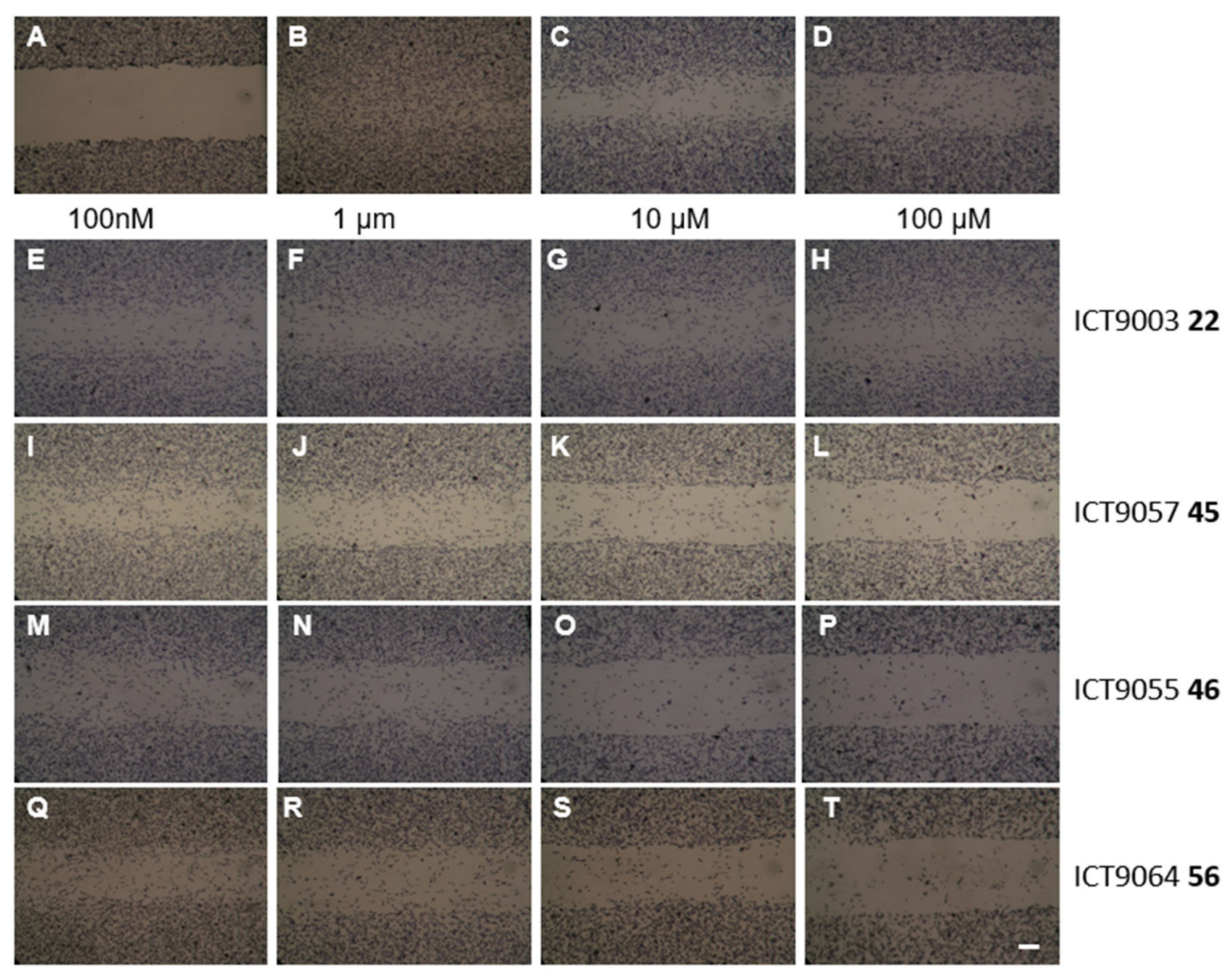
Representative images of the effect of integrin antagonists on the migration of M14 cells in a scratch wound assay. (**A**) Scratch at time 0; (**B**) untreated scratch after 24 h; (**C**) scratch treated with 500 nM cRGDfV positive control after 24 h; (**D**) scratch treated with 5 µM cRGDfV positive control after 24 h; (**E**–**H**) scratches treated with the indicated concentrations of ICT9003 **22** after 24 h; (**I**–**L**) scratches treated with the indicated concentrations of ICT9057 **45** after 24 h; (**M**–**P**) scratches treated with the indicated concentrations of ICT9055 **46** after 24 h; (**Q**–**T**) scratches treated with the indicated concentrations of ICT9064 **56** after 24 h. Bar length = 200 μm.

The anti-αvβ3 activity of selected compounds was confirmed in a wound-healing assay (Figure 3). A number of cell lines were initially investigated for their suitability in this assay. The Sk-Mel-2 cell line which adhered well to fibronectin in the adhesion assay migrated very slowly; therefore, M14 melanoma cells which combined high levels of αvβ3 with rapid migration and low levels of proliferation over the assay period (Supplementary Figure S4) were selected. In general, compounds which were active in the Sk-Mel-2 cell adhesion assay were also effective at preventing M14 cell migration (Table 2 Column 10; Supplementary Figure S5); again, ICT9055 **46** and its free acid ICT9064 **56** were the most active. ICT9062 **49** showed a similar activity but this result must be treated with caution as it was also the most cytotoxic of the compounds and lower in purity than ICT9055.

A selection of compounds were tested for activity against αIIbβ3 to differentiate hit dual β3 antagonists from selective αvβ3 antagonists and also further explore the compound SAR. Activity against αIIbβ3 was initially tested in a cell-free αIIbβ3 protein binding ELISA [71], followed by a testing of selected compounds for the inhibition of ADP-induced platelet aggregation in hirudin-treated whole blood as a physiologically relevant antiplatelet assay (Table 3). Some compounds, notably free acids ICT9090 **53** and ICT9064 **56** were moderately active in vitro but were significantly less active against platelet aggregation ex vivo. *Trans*-cyclobutanes were slightly more effective than *cis*, e.g., *cis* ICT9028 **51** IC_50_ 7.8 μM vs. *trans* ICT9029 **54** 3.3 μM, *cis* ICT9030 **50** 3.5 μM vs. *trans* ICT9031 **52** 1.7 μM, supporting the original molecular modelling that such compounds are slightly longer, thus a better match to the αIIbβ3 binding site. As with anti-αvβ3 activity, the mesitylsulfonamide exosite-binding group conferred higher anti-αIIbβ3 activity; ICT9090 **53** IC_50_ 0.39 μM vs. phenylsulfonamide ICT9029 **54** 3.3 μM.

## 4. Discussion

Metastatic dissemination of melanoma, including haematogenous metastasis, is still a clinically relevant problem despite the introduction of new targeted therapies. The high expression of the β3 subunit [90,91,92,93] and αvβ3 have been reported to be a characteristic of melanoma [94] and the ectopic expression of αIIbβ3 also occurs [29,30]. Therefore, melanoma cell lines were chosen for use in assays of integrin-mediated adhesion and migration as a first step to developing β3 integrin antagonists which could be developed as potential treatments for advanced melanoma. Our results confirmed a high αv and β3 expression in the M14 and Sk-Mel-2 cell lines used for adhesion and migration studies. However, these lines did not express detectable levels of αIIb mRNA or protein; Kopatz and Selzer [95] have also reported αIIb was unquantifiable in a wider panel of melanoma cell lines. It is known that integrin expression alters in response to the extracellular matrix [96], and, in prostate cancer, αIIb expression is present in vivo but reduced by in vitro culture [32].

Biological investigation of this small library of compounds provided structure activity relationship information on the role of the cyclobutane geometry, linker length, and the identity and stereochemistry of the Asp mimetic. THN-containing compounds had higher anti-αvβ3 antiadhesive activity than those containing naphthyridine or pyrimidine Arg mimetics, for example, pyrimidine ICT9020 **24** 19.1% vs. naphthyridine ICT9053 **41** 27.5% and THN ICT9055 **46** 98.4% inhibition of adhesion at 5 μM. Compounds without a sulfonamide exosite-binding group [97,98] showed little activity; the most active example was THN ICT9066 **47**, which inhibited 22.5% of the adhesion at 50 μM. A more lipophilic group increased the activity, for example, phenylsulfonamide ICT9026 **29** was essentially inactive and ICT9057 **45** gave a 59.5% inhibition at 5 μM vs. mesitylsulfonamides ICT9020 **24** 19.1% and ICT9055 **46**’s 98.4% inhibition at 5 μM

The length of the linker had little effect in the pyrimidine series (n = 1 ICT9019 **21** 35.2%; n = 2 ICT9020 **24** 19.1%; n = 3 ICT9018 **26** 20.9% adhesion inhibition at 5 μM), but the shorter THN ICT9080 **48** (61.5%) was less active than ICT9055 **46** (98.4% adhesion inhibition at 5 μM). There was no clear relationship between *cis*- vs. *trans*-substituted cyclobutane rings and antiadhesive activity; three out of five pairs (**21**/**27**; **23**/**29**; **41**/**44**) had no significant difference in activity, and in the other two (**24**/**28** and **46**/**49**), the *cis* was slightly more effective.

Activity in the wound healing assay was less sensitive to the identity of the exosite-binding group; phenylsulfonamide ICT9057 **45** (IC_50_ 1.0 ± 0.09 μM) and ICT9003 **22** (IC_50_ 4.8 ± 0.2 μM) with no exosite-binding group were both significantly more active in this assay than anticipated from their effects on cell adhesion. Apart from this, trends in anti-αvβ3 activity were consistent between assays: THN was the most active Arg mimetic (ICT9055 **46** IC_50_ < 0.1 μM and its free acid ICT9064 **56** IC_50_ 0.2 ± 0.06 μM) and configuration of the cyclobutane had little impact on activity (*trans* THN ICT9062 **49** IC_50_ 0.15 ± 0.03 μM; slightly less active than *cis* ICT9055 **46**). In general, active compounds showed lower IC_50_s for the inhibition of the M14 cell migration than they did for the Sk-Mel-2 adhesion despite the very similar integrin expression profile of the two cell lines. These melanoma cell lines contain different mutations in genes such as BRAF and NRAS [99,100] which activate the same cell signalling pathways as that of the integrin ligation, so this could affect their responsiveness to integrin antagonists. There is a need for further work to establish the effects of nonintegrin receptors and signalling pathways dysregulated in cancer on the response to integrin antagonists; we are working to establish the interactions between integrin and nonintegrin receptors and identify effective combination therapies using integrin antagonists with other targeted therapeutics.

Cell adhesion and migration are both essential processes in the metastatic pathway. β3 integrin function is known to be required for adhesive cell–cell and cell–matrix interactions facilitating the formation of new tumours [101,102,103]. The identification of ICT9055 **46** and the corresponding acid ICT9064 **56** as more effective than positive control cRGDfV in both cell-based assays identified it as a compound of interest, and we progressed to assess its compound activity against αIIbβ3.

**Figure 4:**
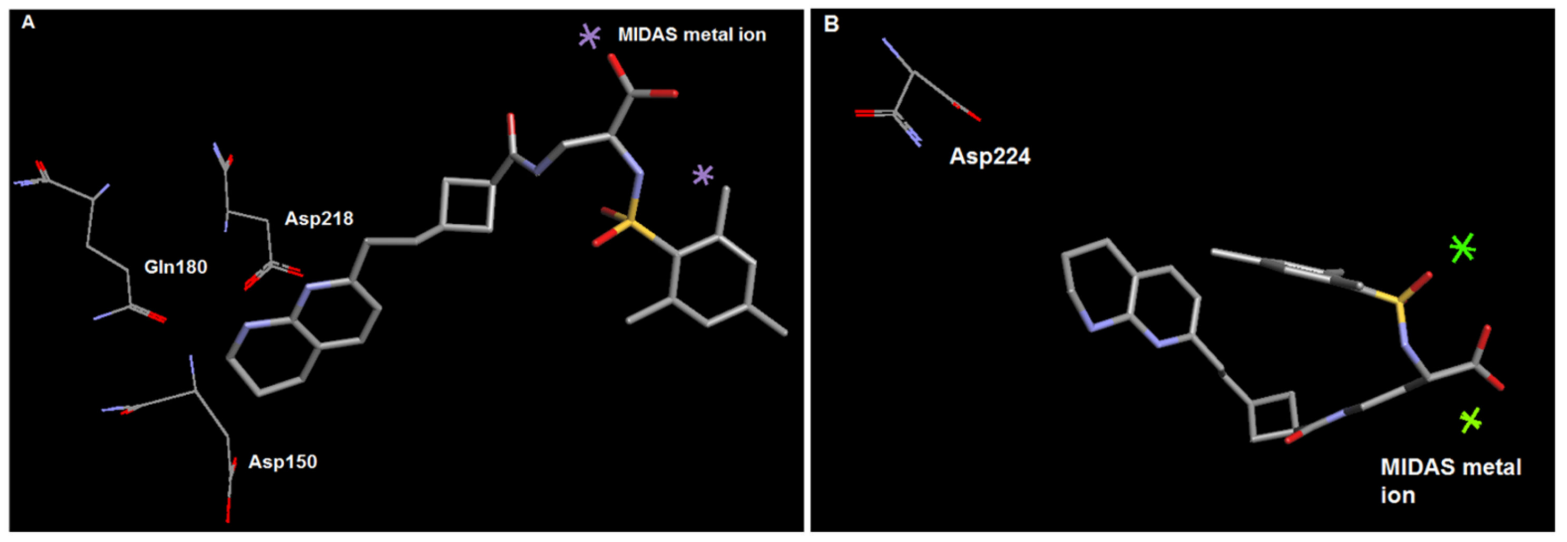
Molecular docking (Arguslab) shows ICT9064 **56** interacts efficiently with key residues in αvβ3 but is not long enough to interact with αIIb Asp224 in αIIbβ3: (**A**) ICT9064 in the ligand-binding site of αvβ3 crystal structure IL5G; (**B**) ICT9064 in the ligand-binding site of αIIbβ3 crystal structure ITY5. Metal ions in the β3 subunit are indicated by (**A**) purple and (**B**) green stars respectively, with the MIDAS site ion labelled.

As expected, esters were less active (all IC_50_ 50 μM or greater) than the corresponding free acids in inhibiting the binding of αIIbβ3 to fibrinogen since they are unable to form ionic bonds to the MIDAS metal ion. Several free acids had a moderate ability to block the αIIbβ3/Fg interaction in vitro (IC_50_ ranging from 0.39 ± 0.19 μM (ICT9090 **53**) to 7.8 ± 2.8 μM (ICT9028 **51**)), however their ability to prevent ex vivo platelet aggregation was much lower (11.1–31.4% inhibition at 100 μM with ICT9064 **56** as the most effective inhibitor). To rationalize the lower anti-αIIbβ3 activity of ICT9064, the compound was docked into the binding sites of αvβ3 (PDB crystal structure IL5G) and αIIbβ3 (PDB crystal structure ITY5) using Arguslab. The lowest energy poses (Figure 4) suggested that ICT9064 could adopt both an extended and a curved conformation; the extended conformation effectively bridges the distance between the αv Asp218 residue and allows for a two-point interaction between this residue and the two THN nitrogen atoms, and the β3 metal ion effectively fills the αv RGD-binding site. However, docking with αIIbβ3 showed binding to the β3 metal ion only with the molecule in a more curved conformation which did not place the THN nitrogen atoms near the αIIb Asp224 residue. This is consistent with the compounds’ observed profile of higher inhibition of functional αvβ3 activity, although it should be interpreted with some caution given the limitations of Arguslab [104]. Additionally, the minimum energy conformation of some compounds was investigated using the inbuilt MM2 function in Chem3D v15. This supported the existence of a low energy curved conformation, giving a distance of only 8.4 Å between the Arg mimetic nitrogen and Asp mimetic carboxylate in ICT9064. However, 8.4 Å is shorter than the αvβ3 binding site as well, which supports other more extended conformations being available for binding.

Despite the lower than anticipated anti-αIIbβ3 activity, the effectiveness of ICT9064 and its prodrug ester ICT9055 identify the 1,3-substituted cyclobutane structure as a starting point for modifying the flexibility of the molecular skeleton to develop higher-affinity compounds which will be investigated in models of melanoma dissemination.

During this project, cyclobutane-containing αv integrin antagonists developed by Bristol Myer Squibb (BMS) were reported in the patent literature. This included the independent synthesis of ICT9064 **56** and related compounds. The IC_50_s of **56** were reported to be αvβ3 2.08 nM; αvβ5 0.2 nM; αvβ6 0.37 nM; αvβ1 3.1 nM and αvβ8 15 nM in a cell-free homogeneous time-resolved fluorescence assay [105]. This activity in a cell-free assay is consistent with the effects on integrin function we observed in high-αvβ3-expressing cells, and taken together, these results indicate the THN arginine sidechain mimetic and mesityl exosite-binding group are important in controlling the binding affinity to αv-subfamily integrins. The BMS methods for synthesising cyclobutane integrin antagonists involve adding sidechains to a small cyclobutane building block, so they are limited by the existing building blocks available. Since our method allows the incorporation of functional groups at all positions of the cyclobutane ring, either by the choice of reactants in the cyclisation step or by a later functionalisation of the cyclobutene, it gives a more flexible approach and will be more suited for synthesizing and refining further potential antagonists for investigation.

## 5. Conclusions

In summary, we have developed a telescoped synthesis of functionalised cyclobutenes from aldehydes, which facilitates the versatile and efficient large-scale synthesis of novel molecules. This methodology can be used to generate cyclobutanes bearing protected amine, alcohol, or carboxylic acid sidechains with both 1,3-*cis* and 1,3-*trans* geometry, thus providing diverse building blocks for further elaboration to integrin antagonists, small molecules targeting other receptors or enzymes, or natural products. We have synthesised a small library of cyclobutane-based RGD-mimetic anti-integrin agents and report the first assessment of these compounds in β3 integrin functional assays. This is the first demonstration that cyclobutanes are effective β3 integrin inhibitors in cancer cell lines and lays the foundation for future development of dual- or singly targeted anti-integrin agents as effective cancer therapeutics.

## 6. Patents

UK patent application No. 2301024.2 avb3 integrin antagonists filed 24 January 2023.

## Figures and Tables

**Table 1:** Summary of final compound structures synthesised and yields of esters from the final step.

Ester	Yield/%	Acid	Arg Mimetic	n	R^1^	R^2^
**21** ICT9019	34.5	**50** ICT9030	Pyrimidine	1	NHSO_2_Mes	H
**22** ICT9003	65	-	Pyrimidine	2	H	H
**23** ICT9023	21	**51** ICT9028	Pyrimidine	2	NHSO_2_Ph	H
**24** ICT9020	18	-	Pyrimidine	2	NHSO_2_Mes	H
**25** ICT9021	28	-	Pyrimidine	2	H	NHSO_2_Ph
**26** ICT9018	59	-	Pyrimidine	3	NHSO_2_Mes	H
**27** ICT9024	38	**52** ICT9031	Pyrimidine ^T^	1	NHSO_2_Mes	H
**28** ICT9025	35	**53** ICT9090	Pyrimidine ^T^	2	NHSO_2_Mes	H
**29** ICT9026	41	**54** ICT9029	Pyrimidine ^T^	2	NHSO_2_Ph	H
**40** ICT9054	38	-	Naphthyridine	1	NHSO_2_Ph	H
**41** ICT9053	28	**55** ICT9063	Naphthyridine	1	NHSO_2_Mes	H
**42** ICT9065	55	-	Naphthyridine	1	H	H
**43** ICT9079	23.5	-	Naphthyridine	0	NHSO_2_Mes	H
**44** ICT9061	27	-	Naphthyridine ^T^	1	NHSO_2_Mes	H
**45** ICT9057	38	-	THN	1	NHSO_2_Ph	H
**46** ICT9055	69	**56** ICT9064	THN	1	NHSO_2_Mes	H
**47** ICT9066	77	-	THN	1	H	H
**48** ICT9080	87.5	-	THN	0	NHSO_2_Mes	H
**49** ICT9062	62	-	THN ^T^	1	NHSO_2_Mes	H

^T^ *trans* configuration of cyclobutane sidechains.

**Table 2:** Anti-αvβ3 activity. Columns 7–9: inhibition of αvβ3 mediated Sk-Mel-2 cell adhesion to Fn at the stated concentration and IC_50_ for selected compounds. Column 10: activity of selected compounds in the M14 scratch wound healing assay. Data are given as the mean ± SD of a minimum of 3 independent experiments.

Compound	Arg Mimetic	n	R^1^	R^2^	Acid/Ester	Adhesion % Inhibition @ 50 μM	Adhesion % Inhibition @ 5 μM	Adhesion IC_50_/µM	Migration IC_50_/µM
ICT9019 **21**	Pyrimidine	1	NHSO_2_Mes	H	Ester	48.3 ± 4.1	35.2 ± 19.1	50 ± 5	9.5 ± 0.9
ICT9003 **22**	Pyrimidine	2	H	H	Ester	^a^	^a^	-	4.8 ± 0.2
ICT9023 **23**	Pyrimidine	2	NHSO_2_Ph	H	Ester	7.4 ± 15.1	^b^	-	-
ICT9020 **24**	Pyrimidine	2	NHSO_2_Mes	H	Ester	-	19.1 ± 14.2	-	>10
ICT9021 **25**	Pyrimidine	2	H	NHSO_2_Ph	Ester	22.2 ± 22.3	^b^	-	-
ICT9018 **26**	Pyrimidine	3	NHSO_2_Mes	H	Ester	-	20.9 ± 27.5	-	>10
ICT9024 **27**	Pyrimidine ^T^	1	NHSO_2_Mes	H	Ester	94.2 ± 4.1	39.4 ± 17.1	13 ± 3	-
ICT9025 **28**	Pyrimidine ^T^	2	NHSO_2_Mes	H	Ester	92.5 ± 8.1	0 ± 19.6	38 ± 11	-
ICT9026 **29**	Pyrimidine ^T^	2	NHSO_2_Ph	H	Ester	92.4 ± 8.0	0 ± 17.3	>50	-
ICT9054 **40**	Naphthyridine	1	NHSO_2_Ph	H	Ester	-	48.7 ± 30.6	-	-
ICT9053 **41**	Naphthyridine	1	NHSO_2_Mes	H	Ester	-	27.5 ± 32.9	>5	>10
ICT9065 **42**	Naphthyridine	1	H	H	Ester	-	^-^	-	-
ICT9079 **43**	Naphthyridine	0	NHSO_2_Mes	H	Ester	-	33.5 ± 26.2	-	-
ICT9061 **44**	Naphthyridine ^T^	1	NHSO_2_Mes	H	Ester	-	32.4 ± 16.1	-	-
ICT9057 **45**	THN	1	NHSO_2_Ph	H	Ester	-	59.5 ± 24.0	-	1.0 ± 0.09
ICT9055 **46**	THN	1	NHSO_2_Mes	H	Ester	-	98.4 ± 1.9	0.34 ± 0.33	<0.1
ICT9066 **47**	THN	1	H	H	Ester	22.4 ± 29.3	^b^	-	-
ICT9080 **48**	THN	0	NHSO_2_Mes	H	Ester	-	61.5 ± 11.1	-	-
ICT9062 **49**	THN^T^	1	NHSO_2_Mes	H	Ester	-	72.3 ± 9.4	-	0.15 ± 0.03
ICT9030 **50**	Pyrimidine	1	NHSO_2_Mes	H	Acid	51 ± 9.0	4.5 ± 19.6	50 ± 7	-
ICT9028 **51**	Pyrimidine	2	NHSO_2_Ph	H	Acid	31.8 ± 10.0	^b^	>50	-
ICT9031 **52**	Pyrimidine ^T^	1	NHSO_2_Mes	H	Acid	11.1 ± 2.4	^b^	>50	-
ICT9090 **53**	Pyrimidine ^T^	2	NHSO_2_Mes	H	Acid	68.9 ± 10.9	-	11.0 ± 11.6	-
ICT9029 **54**	Pyrimidine ^T^	2	NHSO_2_Ph	H	Acid	42.5 ± 14.1	^b^	>50	-
ICT9063 **55**	Naphthyridine	1	NHSO_2_Mes	H	Acid	-	20.7 ± 11.9	>5	-
ICT9064 **56**	THN	1	NHSO_2_Mes	H	Acid	-	76.1 ± 20.4	3.7 ± 2.3	0.2 ± 0.06
cRGDfV						-	61.5 ± 15.1	2.1 ± 0.8	4.0 ± 0.07
RGDS						45.8 ± 16.6	68.5 ± 29.1	41 ± 13	-

^a^ Negligible activity at 10 μM. ^b^ Negligible activity at 5 μM. - Not tested. ^T^ *trans* configuration of cyclobutane sidechains.

**Table 3:** Inhibition of αIIbβ3-fibrinogen interaction. Structurally related compounds are listed together with the ester first then the free acid. αIIbβ3/Fg refers to purified protein ELISA. Data are given as the mean ± SD of a minimum of 3 independent experiments. Platelet aggregation refers to ex vivo inhibition of ADP-induced platelet aggregation in hirudin-treated whole blood. GR144053 positive control.

Compound	Arg Mimetic	n	R^1^	R^2^	% Inhibition (αIIbβ3/Fg) @ 50 μM	IC_50_/μM (αIIbβ3/Fg)	% Inhibition (Platelet Aggregation) @ 100 μM
ICT9019 **21**	Pyrimidine	1	NHSO_2_Mes	H	15.6 ± 14.5	-	-
ICT9030 **50**					91.2 ± 9.6	3.5 ± 1.9	-
ICT9023 **23**	Pyrimidine	2	NHSO_2_Ph	H	10.9 ± 15.4	-	-
ICT9028 **51**					93.6 ± 2.2	7.8 ± 2.8	-
ICT9024 **27**	Pyrimidine ^T^	1	NHSO_2_Mes	H	33.5 ± 2.8	50 ± 5	-
ICT9031 **52**					96.2 ± 6.3	1.7 ± 2.4	11.1
ICT9025 **28**	Pyrimidine ^T^	2	NHSO_2_Mes	H	35.0 ± 11.3	>100	-
ICT9090 **53**					-	0.39 ± 0.19	21.2 ± 7.8
ICT9026 **29**	Pyrimidine ^T^	2	NHSO_2_Ph	H	31.6 ± 10.7	-	-
ICT9029 **54**					100 ± 6	3.3 ± 3.3	-
ICT9053 **41**	Naphthyridine	1	NHSO_2_Mes	H	29.4 ± 17.6	>100	-
ICT9063 **55**					84.5 ± 8.6	4.1 ± 2.2	13.0
ICT9055 **46**	THN	1	NHSO_2_Mes	H	0 ± 9.8	>50	5.6
ICT9064 **56**					88.8 ± 2.6	1.17 ± 0.9	31.4
GR144053					-	23.7 ± 3.1 nM	100 (IC_50_ 240 nM)

- Not tested. ^T^ *trans* configuration of cyclobutane sidechains.

## Data Availability

The data presented in this study are available within the article and supplementary file.

## Supplementary Materials

The following supporting information can be downloaded at: https://www.mdpi.com/article/10.3390/cancers15164023/s1, Figure S1: Expression levels of RGD-binding integrin subunit mRNA in Sk-Mel-2 and M14 cell lines; Figure S2: Expression of integrin subunits in in Sk-Mel-2 and M14 cell lines by Western blot; Figure S3: Quantification of αV and β3 integrin subunits in a panel of human tumour cell lines; Figure S4: Immunocytochemical analysis of M14 cells; Figure S5: Example of analysis of scratch assay; Table S1. Cytotoxicity of compounds on the cell lines used in the functional assays.
