# Supplementary material for: Synthesis and Biological Evaluation of Cyclobutane-Based β3 Integrin Antagonists: A Novel Approach to Targeting Integrins for Cancer Therapy
Source: Cancers (Basel). 2023 Aug 8;15(16):4023. doi: 10.3390/cancers15164023 (PMC10452181; doi:10.3390/cancers15164023)
Supplement: Supplementary file 1 [file cancers-15-04023-s001.zip › cancers-2480439-supplementary.pdf]

Supplementary information

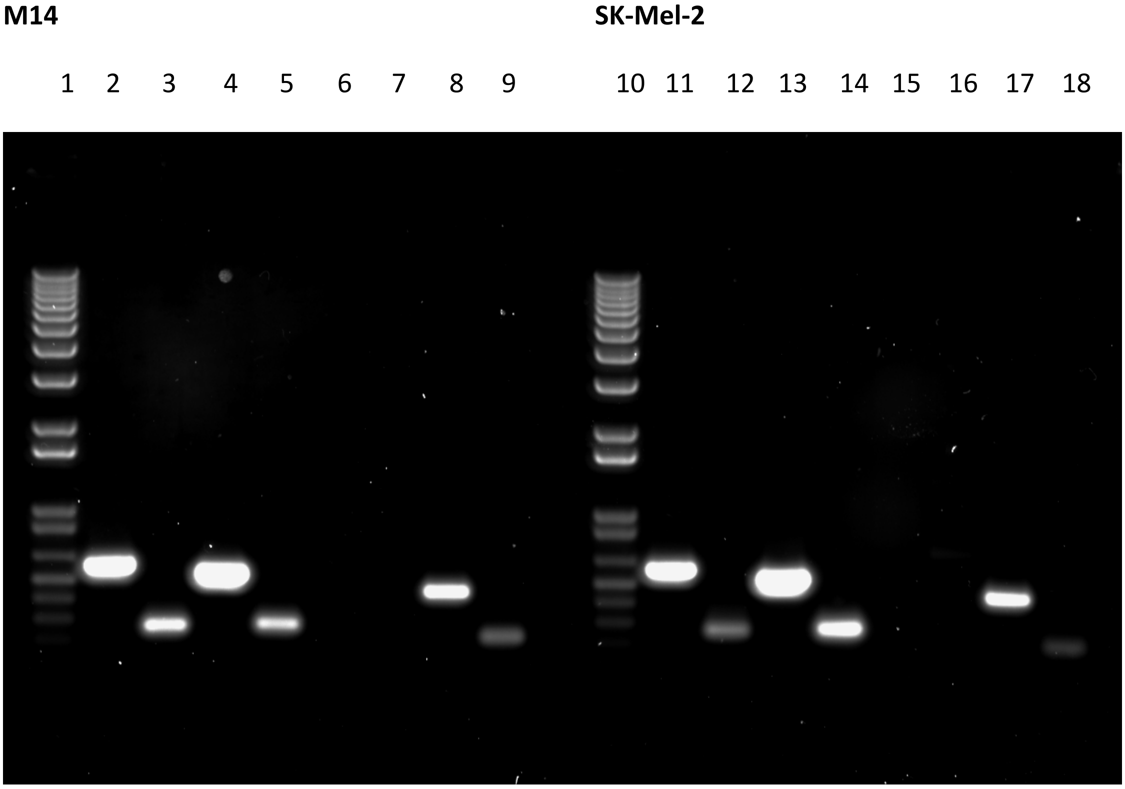

Figure S1: Expression levels of RGD-binding integrin subunit mRNA in Sk-Mel-2 and M14 cell lines. Total RNA was isolated from cell pellets, after first strand cDNA synthesis GAPDH and the integrins were amplified by PCR. Following agarose gel electrophoresis, band intensity was determined using a Molecular Imager FX and Quantity One Software and results expressed as ratio of integrin/GAPDH. Lanes 1 and 10: molecular weight marker; Lanes 2 and 11: GAPDH; Lane 3 and 12: β_1; Lane 4 and 13: β_3; Lane 5 and 14: β_5; Lane 6 and 15: β_6; Lane 7 and 16: α_{IIb}; Lane 8 and 17: α_v; Lane 9 and 18: α5.

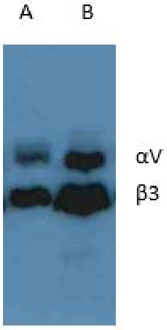

Figure S2: Expression of integrin subunits in Sk-Mel-2 and M14 cell lines by Western blot. 75 µg total lysate of M14 (Lane A) and SK-Mel-2 (Lane B) were separated on a 6% polyacrylamide gel and detected with anti-β_3 monoclonal antibody sc-46655 (Santa Cruz Biotech) and anti-α_v monoclonal antibody Q-20 sc-6617-R (Santa Cruz Biotech).

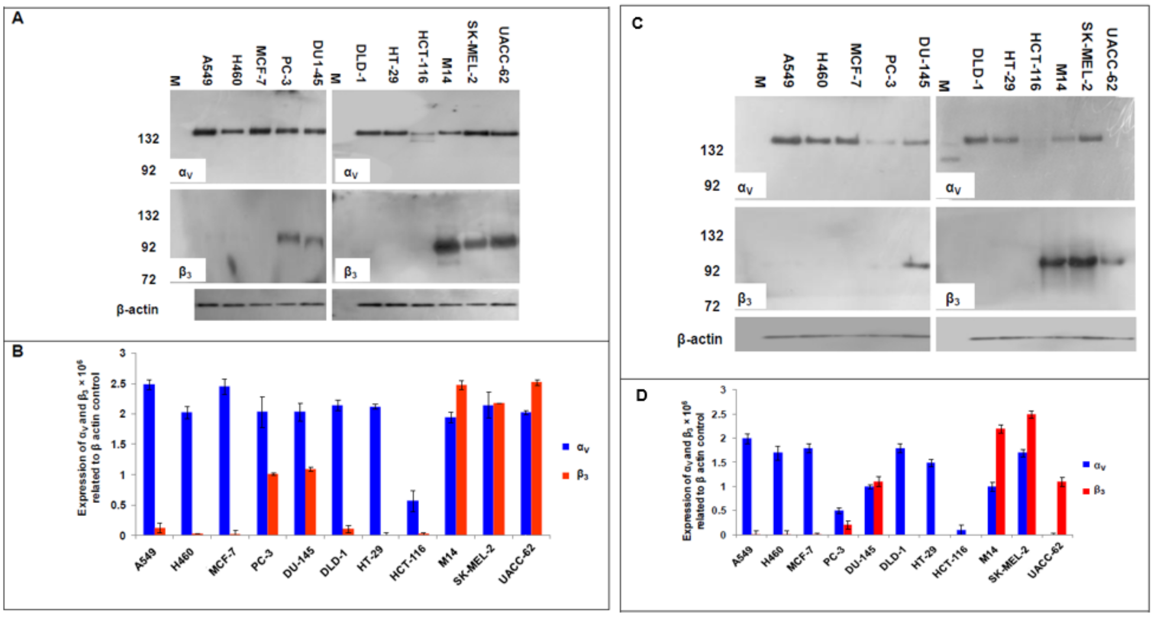

Figure S3: Quantification of αV and β3 integrin subunits in a panel of human tumour cell lines. Prior to Western blotting using the conditions and antibodies in S2, cells were lysed for 1h on ice in lysis buffer (2X lysis buffer [100 mM Tris pH 8.8, 5 mM EDTA, 300 mM NaCl, 2% Triton X-100], protease inhibitor (Complete mini EDTA-free (Roche, Mannheim, Germany) and water), after which sonication (Philip Harries Scientific Sonicator, Scientific Laboratory) was carried out for two pulses of five seconds each. Sonicated cells were centrifuged at 9000 rcf for ten minutes at -20 °C, after which the cytosolic supernatant of the lysed cells was collected and the cellular pellet resuspended in fresh lysis buffer. A: Cytosolic expression of αV and β3. M Lane 1 is Page Ruler Plus Prestained Protein Ladder marker range from 10 to 250 kDa but because of a short exposure time of five minutes it is not clearly seen on the film. C. Plasma membrane expression of αV and β3 integrin subunits from cell pellets resuspended in lysis buffer. B and D. Films were analysed using a Bio Rad Molecular Imager FX. The expression of αV and β3 band density and β-actin band density were subtracted from the film background density. αV and β3 signal intensity were divided by β-actin signal intensity to obtain relative expression levels.

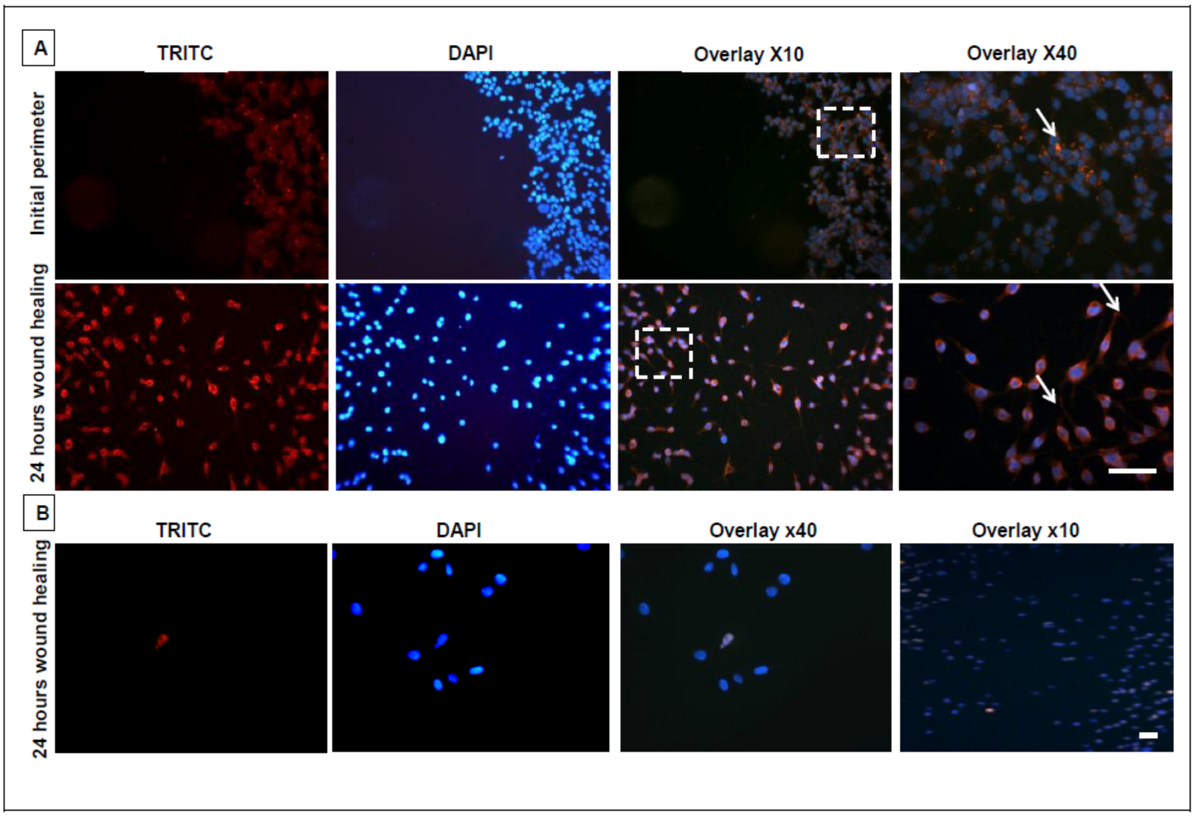

Figure S4: Immunocytochemical analysis of M14 cells at the initial scratch perimeter (t = 0) and after 24 hours of wound healing. A. Expression of $\alpha v\beta_3$ integrin at the initial wound perimeter and after 24 hours of wound healing using LM609, anti-$\alpha v\beta_3$ integrin antibody. All M14 cells expressed $\alpha v\beta_3$ at the initial wound perimeter and also after 24 hours. Cells migrating into the wound area showed $\alpha v\beta_3$ integrin expression as indicated by white arrows. B. The immunolabelling with Ki-67 showed nuclear expression and confirmed that M14 cells moving into the wound area had migrated. Bar length (A) = 60 µm and (B) = 200 µm.

Table S1. Cytotoxicity of compounds on the cell lines used in the functional assays (measured by MTT assay). Cells (2000 cells/well for Sk-Mel-2; 1 x 10^4 cells/mL for M14) were incubated for 96 hours at 37 °C in a total volume of 200 µL/well (180 µL medium + 20 µL test compound solution). After 96 hours, the medium was replaced by fresh medium containing 0.5 mg/ml (end conc.) MTT per well, incubated for 4 h then the plate processed and read as described in the main text.

Compound	Arg mimetic	n	R^1	R^2	Acid/ester	Sk-Mel-2 IC_{50}/µM	M14 IC_{50}/µM
ICT9019 **21**	pyrimidine	1	$NHSO_2Mes$	H	Ester	53.8 ±3.2	>100
ICT9003 **22**	pyrimidine	2	H	H	Ester	>100	>100
ICT9023 **23**	pyrimidine	2	$NHSO_2Ph$	H	Ester	>100	-
ICT9020 **24**	pyrimidine	2	$NHSO_2Mes$	H	Ester	57.8 ± 1.9	36 ± 18.6
ICT9021 **25**	pyrimidine	2	H	$NHSO_2Ph$	Ester	>100	-
ICT9018 **26**	pyrimidine	3	$NHSO_2Mes$	H	Ester	72.0 ± 6.5	>100
ICT9024 **27**	pyrimidine^T	1	$NHSO_2Mes$	H	Ester	55.9 ± 4.9	-
ICT9025 **28**	pyrimidine^T	2	$NHSO_2Mes$	H	Ester	55.3 ± 1.6	-
ICT9026 **29**	pyrimidine^T	2	$NHSO_2Ph$	H	Ester	>100	-
ICT9054 **40**	naphthyridine	1	$NHSO_2Ph$	H	Ester	65.2 ± 1.3	-
ICT9053 **41**	naphthyridine	1	$NHSO_2Mes$	H	Ester	52.6 ± 0.6	72.7 ± 9.8
ICT9065 **42**	naphthyridine	1	H	H	Ester	>100	-
ICT9079 **43**	naphthyridine	0	$NHSO_2Mes$	H	Ester	~100	-
ICT9061 **44**	naphthyridine^T	1	$NHSO_2Mes$	H	Ester	46.8 ± 10.7	-
ICT9057 **45**	THN	1	$NHSO_2Ph$	H	Ester	65.4 ± 11.1	>100
ICT9055 **46**	THN	1	$NHSO_2Mes$	H	Ester	9.6 ± 5.7	>100
ICT9066 **47**	THN	1	H	H	Ester	>100	-
ICT9080 **48**	THN	0	$NHSO_2Mes$	H	Ester	54.6 ± 11.0	-
ICT9062 **49**	THN	1	$NHSO_2Mes$	H	Ester	>5	3.3 ± 0.8
ICT9030 **50**	pyrimidine	1	$NHSO_2Mes$	H	Acid	90.2 ± 4.3	-
ICT9028 **51**	pyrimidine	2	$NHSO_2Ph$	H	Acid	>100	-
ICT9031 **52**	pyrimidine^T	1	$NHSO_2Mes$	H	Acid	>100	-
ICT9090 **53**	pyrimidine^T	2	$NHSO_2Mes$	H	Acid	>50	-
ICT9029 **54**	pyrimidine^T	2	$NHSO_2Ph$	H	Acid	>100	-
ICT9063 **55**	naphthyridine	1	$NHSO_2Mes$	H	Acid	61.7 ± 2.8	-
ICT9064 **56**	THN	1	$NHSO_2Mes$	H	Acid	45.5 ± 7.9	>100
cRGDfV						42.5 ± 1.8	>50
RGDS						>50	-

^{T} *trans* configuration of cyclobutane sidechains.

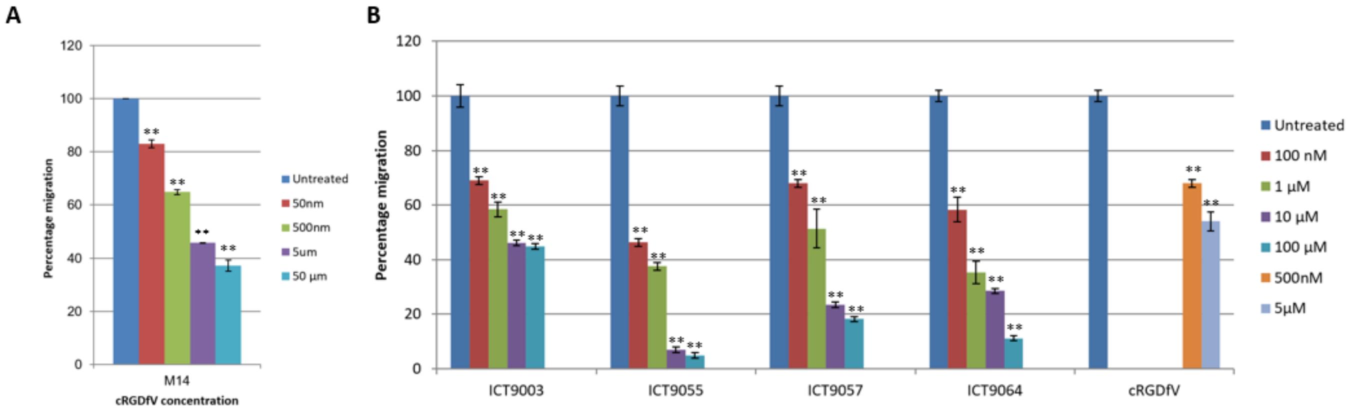

Figure S5. Example of analysis of scratch assay. A Effect of cRGDfV on M14 cell migration compared vs untreated cells. B Effect of ICT9003, ICT9055, ICT9057 and ICT9064 on M14 cell migration at the indicated concentrations. cRGDfV at 0.5 and 5 µM was included in each run as positive control. Data are presented as mean ± standard deviation, with each experiment repeated at least three times. The student's t-test (parametric) and kruskall wallis (non parametric) test were used for statistical analysis of the scratch assays, with results considered statistically significant and highly significant for $p < 0.05$ (*) and $p < 0.01$ (**) respectively.
